# Landscape and Climate‐Associated Selection in the Native and Widespread Bumblebee, *Bombus terrestris*

**DOI:** 10.1111/mec.70141

**Published:** 2025-10-09

**Authors:** Cecilia Kardum Hjort, Rachael Y. Dudaniec, Peter Olsson, Johan Ekroos, Henrik G. Smith

**Affiliations:** ^1^ Department of Biology Lund University Lund Sweden; ^2^ School of Natural Sciences Macquarie University Sydney New South Wales Australia; ^3^ Centre for Environmental and Climate Science Lund University Lund Sweden; ^4^ Department of Agricultural Sciences Helsinki University Helsinki Finland; ^5^ Helsinki Institute of Sustainability Science, HELSUS University of Helsinki Helsinki Finland

**Keywords:** agriculture, *Bombus terrestris*, gene flow, landscape simplification, morphology, neutral genetic divergence, selection

## Abstract

Anthropogenic land‐use and climate change pose novel selection pressures on bees, yet their evolutionary responses in terms of morphological or physiological adaptations remain unclear. While adaptive responses are expected, these may be constrained by gene flow when changes in selection pressures are spatially heterogeneous. The buff‐tailed bumblebee ( *Bombus terrestris* ) is a widespread species that copes well with anthropogenic land‐use and climate change, suggesting high adaptive capacity or phenotypic plasticity. Here, we genotyped populations of native *B. terrestris* in south and central Sweden using RADseq to investigate genetic structure and local adaptation across a paired design of agricultural landscapes with high and low land‐use complexity along a geographic climate gradient. We expected to find genetic structure reflective of regional barriers to gene flow, and molecular evidence for local adaptation to differing landscape and climate conditions. We found genetic structure separating southern Sweden from more northern regions, with a negative Tajima's *D* indicating a potential population expansion, likely northwards and inland into forested areas, consistent with observational data indicating a range shift. We found weak but significant evidence for local adaptation to climate and land use, specifically to agricultural land cover, including genes under putative selection linked to insecticide resistance. Signatures of selection were also identified in relation to latitude, temperature, and urban land cover, with other candidate SNPs associated with olfaction and immune response. Our results suggest that *B. terrestris* successfully responded to anthropogenic land‐use and climate changes, likely due to its generalist traits, enabling phenotypic adaptation to changing environments.

## 1 Introduction

Understanding how wild bees will respond to anthropogenic land‐use and climate change is crucial for maintaining the functioning of both natural and agricultural ecosystems, given the role of bees as pollinators of wild plants and crops (Klein et al. 2007; Ollerton et al. 2011). Agricultural change, particularly intensification, is considered a major driver of bee declines in Europe and North America (Dicks et al. 2021; IPBES 2016). Agricultural intensification results in the loss of flower resources because of a loss of semi‐natural habitats (Carvell et al. 2006; Langlois et al. 2020), legume flowers (Bommarco et al. 2012), and flowering weeds (Bretagnolle and Gaba 2015). The effects of climate change, including both gradual changes and more frequent extreme weather events, also contribute to bee declines (Descamps et al. 2021; Rasmont and Iserbyt 2012; Soroye et al. 2020). While climate‐induced range shifts may cause bee species to decline where conditions are no longer suitable (Schweiger et al. 2010), they may expand elsewhere (Kerr et al. 2015).

The ability of bees to withstand anthropogenic pressures including climatic change is influenced by their functional traits (Kazenel et al. 2024; Pardee et al. 2022; Pontarp et al. 2023). For example, agricultural intensification may increase spatial and temporal mismatches between the availability of flower resources and nesting sites for bees (Jachuła et al. 2021; Smith et al. 2014). As a result, only those bee species capable of foraging over larger areas and access the remaining scattered flower resources may persist (Greenleaf et al. 2007; Kremen and M'Gonigle 2015). Furthermore, bees differ in their heat tolerance, and larger species, such as bumblebees (genus *Bombus*), may be more vulnerable to higher temperatures due to slower convective heat loss (Bartomeus et al. 2013; Heinrich and Heinrich 1983; Martinet et al. 2021).

Functional traits can in theory be modified through either phenotypic plasticity or genetic adaptation (Maebe et al. 2021; Pontarp et al. 2023). Phenotypic plasticity refers to the ability of an organism to adjust its traits in response to environmental factors, often within a single generation (Bonamour et al. 2019). For example, plasticity in foraging behaviour or heat tolerance can help species cope with changing conditions (Bonamour et al. 2019). Multi‐generational changes in body size due to habitat fragmentation or heat‐stress tolerance in response to climate change suggest the potential of rapid evolutionary change in bees to anthropogenic pressures (Gérard et al. 2020; Nooten and Rehan 2020; Oliveira et al. 2016; Zhao et al. 2021). However, genetic evidence of such changes is limited. Selection on candidate genes related to physiology, such as heat tolerance (Pimsler et al. 2020; Theodorou et al. 2018) has been observed in response to environmental pressures, while no studies have linked genetic changes in body size. While evolutionary responses to agricultural pressures, such as insecticide resistance (Chakrabarti et al. 2018; Colgan et al. 2022; Hart et al. 2022) and through genes involved in detoxification (Tsvetkov et al. 2023), have been documented, the impact of land‐use and climate change on genetic evolution remains poorly understood.

The potential for evolutionary adaptation to anthropogenic changes may be constrained by gene flow, which generally acts as two opposing forces: either by homogenising or ‘swamping’ adaptive genetic variation via the influx of maladapted or neutral alleles (Wadgymar et al. 2019), or by increasing the chances of local adaptation by adding new genetic material on which selection can act upon (Buckley and Bridle 2014). For bee species where gene flow is often high and genetic structure low, such as bumblebees (Colgan et al. 2022; Heraghty et al. 2023), local genetic adaptation to anthropogenic environmental and climatic change may be weak. However, in the face of gene flow, local adaptation is still possible under strong local selection pressures (Theodorou et al. 2018; Yadav et al. 2019; Kardum Hjort, Paris, et al. 2023).

Furthermore, the effect of gene flow on local adaptation may depend on the type and scale of environmental change. For example, large‐scale climatic changes may affect broad areas simultaneously, and thus, gene flow may not severely constrain local adaptation. However, also spatially heterogeneous agricultural intensification may invoke evolutionary responses, either if selection pressures are strong or landscape fragmentation constrains dispersal. Under such circumstance, differences in environmental conditions may play a significant role in shaping how populations adapt to these local landscapes (Heraghty et al. 2023; Jackson et al. 2018).

During the last century, Sweden's agricultural landscape has undergone dramatic structural changes, including an increase in field sizes driven by major modernisation and agricultural intensification. These changes were further fuelled by the widespread use of fertilisers and pesticides (Björklund et al. 1999). Simultaneously, the climate in Sweden has progressively warmed, with rising mean annual temperatures recorded over recent decades (Alexandersson and Eggertsson Karlström 2001). Together, these shifts in land use and climate have likely altered the environmental conditions that affect many bee species.

The buff‐tailed bumblebee is one of Sweden's most common bumblebee species and plays a vital role in supporting the pollination of many natural and agricultural ecosystems worldwide (Dafni et al. 2010; Goulson 2003). *Bombus terrestris* is a large, mobile, nutritional generalist (Mossberg and Cederberg 2012; Rasmont et al. 2008). It is resilient to variable climatic conditions, including heat waves (Martinet et al. 2021), and cold snaps (Owen et al. 2013), and is often found in open areas dominated by arable fields (Herbertsson et al. 2021). Unlike many other bee species, *B. terrestris* appears to have benefitted from anthropogenic land use and climate change, showing an expansion in its distribution in response to increasing mean annual temperatures (Martinet et al. 2015) and an increase in relative abundance compared to other bumblebee species (Herbertsson et al. 2021). This success may be due to the species' generalist traits, which make it well‐suited to endure land‐use changes and benefit from the increased cultivation of mass‐flowering crops.

Although agricultural change constitutes an appealing testbed for studying how the combined effects of land use and climate change affect pollinators through plastic and evolutionary responses, there is often a lack of historical data to do this (Byers 2017). A more feasible way to study the consequence of agricultural change is using space‐for‐time substitution approaches where spatial differences are assumed to reflect temporal changes (Jackson et al. 2020; Pickett 1989). This approach, which creates contrasts between landscapes with different degrees of agricultural land use and climate, has been widely used in several different disciplines and has recently been applied to the study of evolutionary processes (Wogan and Wang 2018). Although there are limitations to this approach, when used with care it is a robust way to study phenomena that otherwise materialise over large temporal scales (Wogan and Wang 2018). In addition, with the addition of high‐resolution DNA sequencing data, we can study historical processes by looking at contemporary genetic variation, allowing for the detection of selection in local environments (Lowry et al. 2017), partly overcoming some of the constraints of space‐for‐time substitution studies.

Here we investigate molecular evidence for selection in *B. terrestris* that may indicate local adaptation in relation to agricultural land use and climate, while also exploring how gene flow might constrain patterns of selection. Given the spatial mosaic of homogenous and heterogeneous agricultural landscapes in Sweden, and documented evidence of adaptation to land use change over small distances (e.g., Theodorou et al. 2018; Eggenberger et al. 2019) and that habitat loss may limit gene flow (e.g., Jha and Kremen 2013), we hypothesise that the genetic structure of *B. terrestris* populations will show patterns of adaptation to varying landscape characteristics. Specifically, we anticipate observing local and regional differences in genetic structure that reflect adaptation to both land‐use complexity and climatic factors, such as temperature variation across latitude. To test this, we employ a geographically diverse sampling design for *B. terrestris* that contrasts simplified agricultural landscapes with latitudinal variation in max annual temperatures. Through this approach, we aim to: (i) explore the genetic structure of *B. terrestris* populations to identify potential barriers to gene flow linked to land use and climate, and (ii) uncover molecular signatures of adaptive evolutionary responses along a latitudinal and climatic gradient.

## 2 Methods

### 2.1 Landscape and Climate Study Design

**FIGURE 1:**
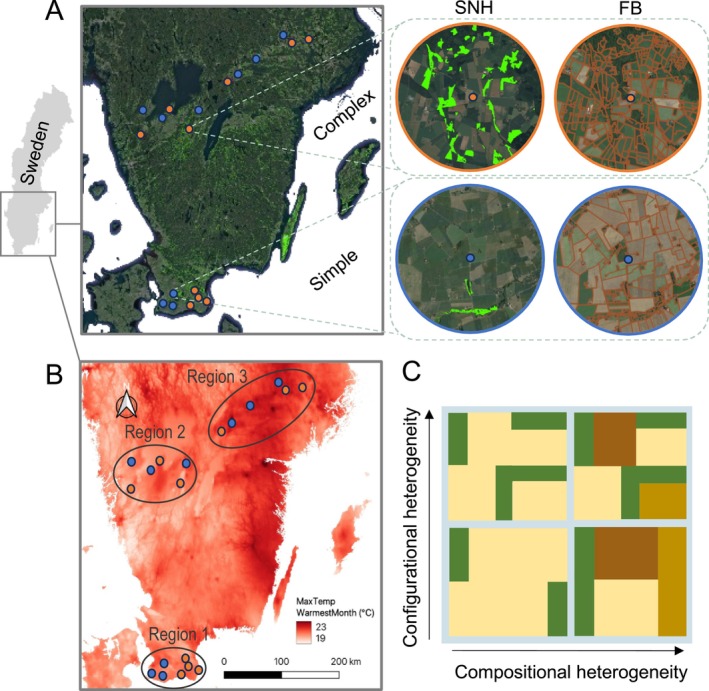
Geographical landscape and climate study design, showing (A) the 19 sampling sites where nine sites are simple (blue) and 10 sites complex (orange). The zoomed‐in circles show semi‐natural habitat (SNH, light green) and uncultivated agricultural field borders (FB, brown lines) for simple respectively complex landscapes; (B) showing the maximum temperature of the warmest month (°C, MaxTempWarmestMonth) across the study design and study regions 1–3; (C) landscape heterogeneity is shown as differences in composition and configuration of agricultural fields (beige and brown) and SNH habitats (green).

The study design was based on 19 study sites, organised into nine matched pairs based on landscape complexity (*n* = 9), in addition to one unmatched study site. Within each pair, landscapes were characterised by either low or high availability of open semi‐natural habitat (simple vs. complex landscapes, respectively), and study site pairs were arranged along a temperature gradient spanning southern and central Sweden (Figure 1A,B).

We defined landscape complexity of the study sites based on the percentage of semi‐natural habitats (SNH, defined as semi‐natural pastures and semi‐natural meadows) and the length of uncultivated agricultural field borders within a circular area of 28 km^2^ (radius 3 km) around their central coordinates. High and low percentages of SNH were intended to reflect compositional landscape changes (Figure 1C, bottom panel) (Fahrig et al. 2011) in the availability of important habitats with flowers and nesting habitat for bees. The length of uncultivated agricultural field borders was in turn intended as a proxy for agricultural field size, reflecting both habitat availability and fragmentation, where longer field borders reflect smaller fields, in relation to the amount of arable land (Figure S1c). Complex landscapes and simple landscapes differed in the amount of semi‐natural habitats (simple: 10–19 ha, complex: 129–195 ha) and length of uncultivated field borders (simple: 190–260 km; complex: 280–450 km) (Figure 1C, top right and top left; Table S1; Figure S1a,b).

Study site pairs were matched to represent similar temperatures across the temperature differences of the geographical gradient (from the southernmost to northernmost pairs; Figure 1B). The design crossed three geographical regions in Sweden: Skåne (region 1), Västra Götaland (region 2) and Mälardalen (region 3) (Figure 1).

In the study site selection, we focused on landscapes with high availability of agricultural land and ensured that complex and simple landscapes had approximately the same agricultural land cover (ranging between 41% and 89%) (Figure S1c). The variable uncultivated agricultural field borders were extracted from the Swedish Integrated Administration and Control System data from 2008 (provided by the Swedish Board of Agriculture), where field borders explicitly represented non‐crop field borders. The variable ‘SNH’ was extracted from the Swedish Land Parcel Identification System (LPIS) data from 2018 (provided by the Swedish Board of Agriculture) supplemented by the Nationella Marktäckedatabasen (NMD, Naturvårdsverket, Stockholm, 2019) for areas not covered by LPIS. The temperature gradient was determined based on the maximum temperature of the warmest month (°C, hereafter ‘MaxTempWarmestMonth’) (Tables S1 and S2), averaged between 1970 and 2000 from WorldClim v2.1and with a resolution of 1 km^2^ (Fick and Hijmans 2017).

### 2.2 Other Environmental and Climate Variables

We extracted information for environmental variables previously suggested to be important to the abundance and distributions of several *Bombus* species to establish gene–environment associations (Svensson et al. 2000; Rasmont et al. 2008; Penado et al. 2016; Kardum Hjort, Paris, et al. 2023; Kardum Hjort, Smith, et al. 2023). Thus, the following additional variables were extracted from WorldClim v2.1 and averaged for 1970–2000: mean annual temperature (hereafter ‘MeanAnnualTemp’ in°C), mean annual precipitation (‘MeanAnnualPrecip’ in mm), precipitation seasonality (‘SeasonPrecip’, the difference between the wettest and driest month, measured as a percentage) and averaged values for June to August of the monthly variables of mean annual wind speed (m/s) (‘AvgSummerWind’ in m/s). Because urban areas may provide resources and forests may be avoided (Lye et al. 2012; Osborne et al. 2008; Svensson et al. 2000) by *B. terrestris*, the variables percentage of urban land cover and the percentage of forest land cover were extracted from the Nationella Marktäckedatabasen (NMD) data. As vegetation height can reflect forest management practices and may explain bumblebee distributions (Geue and Thomassen 2020), canopy height (m) was extracted from the ETH Global Sentinel‐2 10 m Canopy Height (ETHs 2020) database for the year 2020 (Lang et al. 2022) (Table S2).

All environmental variables were extracted from raster layers in R in the 28 km^2^ (radius 3 km) circular areas around the central coordinates. For all environmental and climatic variables (except for the length of agricultural field borders), cell values within the 28 km^2^ area were averaged using weighted mean by cell fraction within the area. The extracted environmental variables were then transformed from cell fraction to percentage of the total 28 km^2^ area. An exception was the agricultural field border, which was measured as all border lengths within the 28 km^2^ area.

### 2.3 Collection and Measuring of *B. terrestris*

We collected *B. terrestris* workers during two time points at each study site in the summer of 2019 (once in June and once in July). *Bombus terrestris* workers were sexed both in the field and subsequently confirmed in the lab by counting the number of segments on the flagellum of the antennae under a dissecting microscope (males 11 segments, females 10 segments after the pedicel). Only female worker bees were retained in the study.

Bumblebees were captured using handheld entomological sweep nets at pre‐existing flower‐rich habitats such as linear elements (e.g., road verges) and semi‐natural vegetation (e.g., pastures and meadows) at different locations within the 28 km^2^ circle and between the two time points. Up to 20 workers were collected per study site and visit, and each collector spent up to 60 min collecting. If after 60 min 20 workers had not been collected, an additional 15–30 min were spent to collect workers. The workers were subsequently placed into 5 mL plastic tubes and kept in a 5°C cooling box to induce chill coma (MacMillan and Sinclair 2011). All bumblebees were then euthanised by placing each 5 mL plastic tube at −20°C for a maximum of 3 h before preservation in 70% ethanol.

*Bombus terrestris* workers were measured for a selection of morphological features: body length (mm), body weight (g), inter‐tegular distance (ITD) (mm), length and area of one small and large wing (mm and mm^2^, for total wing area one wing pair were multiplied by two). Wing loading (body weight in grams divided by the total wing area of four wings in cm^2^) was also calculated (Table S1, detailed description in Text S1).

### 2.4 Distribution of *B. terrestris* in Sweden

Observational occurrence data for *B. terrestris* spanning the period 1970–2023 were obtained from the Global Biodiversity Information Facility (GBIF). The initial year of *B. terrestris* observations were mapped to illustrate its potential range expansion across Sweden using the tMap R package.

### 2.5 CO1 Barcoding and RAD Sequencing

DNA was extracted from one leg of each bumblebee using a custom HotSHOT protocol (see Text S2). To confirm species identification the COI mitochondrial gene was amplified using the protocol of Wahlberg and Wheat (2008) (Text S3). The PCR product was submitted to Macrogen Europe (https://dna.macrogen‐europe.com/) for Sanger sequencing. Each generated COI sequence was uploaded to GenBank Nucleotide BLAST and compared to both complete and partial mitochondrial genome sequences from *B. terrestris* , using a 100% match rate.

Samples identified as *B. terrestris* were re‐extracted for DNA from the head and two legs and prepared into genomic libraries for RAD sequencing at the Diversity Arrays Technology sequencing (DArTseq) facility (Canberra, Australia, Text S4). RAD library preparation was performed by DArTseq following the sequencing protocol described in Kilian et al. (2012) and libraries were run on an Ilumina Hiseq2500. DArT repeats sequencing was performed on approximately 25% of the samples to check for error and allelic dropout. DNA sequences were quality filtered and aligned to the *B. terrestris* reference genome (Sadd et al. 2015) (assembly accession: GCF000214255.1) via BLAST with an *E*‐value of 5 × 10^−7^ and a minimum % of sequence identity of 80. The sequences were also aligned to bacterial and fungal genomes (NCBI), to check for contamination. SNP calling, quality checking, and initial filtering were performed by DArT following the protocol described in Kilian et al. (2012).

### 2.6 SNP Filtering

Sequences were further filtered using the *dartR* package (Mijangos et al. 2022) in R v4.3.1 (R Core Team 2022) to characterise polymorphic SNPs. Monomorphic SNPs were removed, and the dataset was filtered for a depth of coverage between 10 and 200, followed by filtering of call rate by loci of > 85% and removal of SNPs with < 100% reproducibility. A minor allele frequency (MAF) filter of > 0.01 was applied to reduce potential sequencing errors, resulting in dataset 1 (selection detection analyses) and 6702 SNPs. To create dataset 2 (neutral structure analyses), SNPs were further filtered for being out of Hardy Weinberg (HWE), *p*‐value threshold of 1e‐6 using *Plink v.1.9* (Purcell et al. 2007) and removed from the dataset. Lastly, SNPs were filtered for linkage disequilibrium using the—*indep‐pairwise 50* (window size), *5* (step size), and *0.5* (*r* ^2^ threshold) option in *Plink v.1.9* (Purcell et al. 2007), resulting in 4546 SNPs.

Before further analyses, we identified and removed highly related individuals (i.e., full‐siblings) by using the—*relatedness2* filter in VCFtools, based on the methods of Manichaikul et al. (2010) (1st‐degree sibling: 0.177–0.354). In total, we identified seven full‐sibling duos (in sites 460, 784, 4425, 2402 and 5811) (relatedness: 0.302–0.358) and one full‐sibling trio (in site 5534) (relatedness: 0.323–0.330). One individual per sibling pair and two from the sibling trio with the highest percentage of missing data were removed. One individual was further removed from the dataset due to a high percentage of missing data (25%). The final two datasets consisted of 304 individuals (Table S2).

### 2.7 Genetic Diversity and Structure

Using dataset 2 (neutral data), we calculated expected (*H*_E_) and observed (*H*_O_) heterozygosity, allelic richness (*A*_R_), the inbreeding coefficient (*F*_IS_) and pair‐wise *F*_ST_ (Weir and Cockerham 1984) for each site using *hierfstat* v.0.5.11 (Goudet 2005). The statistical significance of *F*_IS_ and *F*_ST_ was based on CIs not overlapping zero (lower 5% and upper 95%) with 10,000 bootstraps. Tajima's *D* was calculated in 10kbp windows for each site using VCFtools to assess if the frequency of alleles is evolving under neutral assumptions. Isolation‐by‐distance (IBD) was assessed using Nei's genetic distance and a Mantel test of 10,000 permutations within the *adegenet* package.

Genetic structure was assessed with a Principal Component Analysis (PCA) and a Discriminant Analysis of Principal Components (DAPC), using the *adegenet* package (Jombart and Ahmed 2011). In addition, three separate PCAs were run on the samples from regions 1–3 to detect the presence of commercial *B. terrestris* , which is known to be genetically distinct in the region (Kardum Hjort et al. 2022). Before running the DAPC, the optimal number of PCs to retain in the analysis was obtained by cross‐validation. To assess individual genomic ancestry and the number of genetic clusters in the dataset (*K*), ADMIXTURE v.1.3.0 (Alexander and Lange 2011) was run with a *K*‐value between 1 and 5 and a 10‐fold cross‐validation (CV) to find the optimum number of genetic clusters. Lastly, fineRADstructure (Malinsky et al. 2018) was used on dataset 1, which uses the whole haplotype to identify genetic structure compared to single SNPs. Using *RADpainter* (Malinsky et al. 2018), the co‐ancestry matrix was calculated and subsequently used to assign each individual to a population by running fineRADstructure with a 100,000 burn‐in period and 100,000 MCMC steps occurring every 1000 MCMC steps.

### 2.8 Effective Migration and Diversity

To visualise spatial genetic structure and migration we implemented the EEMS method (Estimated Effective Migration Surface) (Petkova et al. 2015) on dataset 2, which uses a stepping‐stone model based on calculating resistance distances (i.e., Isolation‐by‐distance, IBD), where individuals migrate between pre‐defined subpopulations (i.e., demes) to generate historic effective migration rates and diversity from geo‐referenced genetic samples. In the model, the area entailing the sampling sites is covered by a dense grid to capture continuous population structure. To reach convergence (checked with the posterior trace plot), EEMS was run three independent times using the *runeems_snps* script with 400,000 MCMC iterations after a burn‐in of 100,000 iterations, and a thinning interval of 9999 between iterations. The deme size (i.e., subpopulations) was set to 600 in all three runs. To generate the final surfaces of effective migration (*m*) and effective diversity (*q*) plots, the results from the three independent runs were combined and plotted using the R package rEEMSplots v 0.0.1 (Petkova et al. 2015).

### 2.9 Detecting Selection Signatures

We first explored possible correlations among the landscape and climate design variables (% SNH, latitude, uncultivated agricultural field borders, and MaxTempWarmestMonth) and among the other environmental and morphological variables separately using *pairs. panel* within the R package *psych*. Uncultivated agricultural field borders and percentage SNH correlated highly (*r* = 0.81) (Figure S2), thus a PCA was run on the two variables and the first PC axis was used as a predictor variable (hereafter ‘PC1_SNH_FB’). The remaining predictors for the landscape and climate design analyses were latitude, PC1_SNH_FB, and MaxTempWarmestMonth (Table S3).

Variable correlations were also explored among the other environmental and morphological variables separately, and variables with correlations of *r* ≥ 0.70 were removed from the analysis (Figure S3), resulting in four environmental variables (MeanAnnualPrecip, SeasonPrecip, percentage urban land cover, and percentage agricultural land cover) and three morphological (ITD, body length, and wing loading) variables and remaining for analysis (Table S3).

### 2.10 LFMM Analysis

We performed a univariate Latent Factor Mixed Modelling analysis (LFMM) (Frichot et al. 2013) with dataset 1 using the *lfmm* package in R (Caye et al. 2019) to investigate potential gene–environment and gene‐morphology associations. Missing genotypes were first imputed, using ancestry coefficients (*snmf* program), using the *impute* function within the *LEA* package (Frichot and François 2015). To find the best numbers of latent factors (i.e., *K*) to include in the LFMM analysis, we ran a PC1_SNH_FB using the *prcomp* function to identify any genetic structure. Subsequently, using *K* = 2, we ran the *lfmm_ridge*, *lfmm_test*, and *calibrate = gif* functions within the *lfmm* package for the three morphological variables (ITD, body length and wing loading) and seven environmental variables (latitude, PC1_SNH_FB, MaxTempWarmestMonth, MeanAnnualPrecip, SeasonPrecip, percentage urban land cover and percentage agricultural land cover). The Genomic Inflation Factor (GIF) was calculated for each predictor variable from the *z*‐scores obtained from the LFMM analysis and assessed in terms of closeness to the recommended value of 1.0. The GIF values ranged between 1.01 and 1.09 and were adjusted, using *adj.pvals1 = pchisq(zscore1^2/new.gif1)* (Forester et al. 2018), if necessary. By subsequently applying the Benjamini–Hochberg procedure (i.e., < 1% FDR), highly correlated SNPs were retained.

### 2.11 RDA Analysis—Landscape and Climate Design

We ran a multivariate Redundancy Analysis (RDA) where the genetic structure is not accounted for, using dataset 1 on the landscape and climate sampling design variables (latitude, PC1_SNH_FB, and MaxTempWarmestMonth). We investigated the Variance Inflation Factor (VIF, a measure of the degree of multicollinearity) using the R package *vegan* (Oksanen et al. 2013) among the variables. The VIF value was < 5 among all three variables (Table S4) and all three variables were included in the RDA analysis, which was run using the *rda* function within the *vegan* package.

### 2.12 RDA Analysis—Environmental and Morphological Predictors

We also ran RDA on the other environmental and morphological variables and found that the variable ‘SeasonPrecip’ had a VIF value of 6.57, indicating moderate multicollinearity with the other predictor variables, and therefore was excluded (Table S4). The RDA was run with three morphological variables and three environmental variables using the *rda* function within the *vegan* package (Table S4). For both RDA analyses, we assessed the number of significant axes to retain for outlier analysis from the full RDA models, running a permutations test (999 replications) within the *anova.cca* function. The final candidate outliers from the two RDA models were identified from the mean SNP loadings (*p* < 0.01).

### 2.13 Gene Annotations

All identified outlier SNPs (i.e., from the two RDA models) were annotated to the *B. terrestris* reference genome using the Burrows‐Wheeler Aligner (BWA‐mem) v0.7.17 (Li and Durbin 2009). The aligned reads were subsequently intersected with the reference genome annotation file, and the gene IDs as well as the start and end positions were extracted to create a reference list of genes. The gene names and potential functions of the genes were identified using the National Centre for Biotechnology Information (NCBI) gene information database. In addition, the genes were annotated against the *B. terrestris* reference genome using the Ensembl Variant Effect Predictor tool (VEP) in the EnsemblMetazoa database to identify any protein‐coding genes and the type of effect (e.g., intron, missense variant, and synonymous variant).

## 3 Results

### 3.1 Genetic Diversity and Differentiation

The observed heterozygosity (*H*_O_) was lower than expected (*H*_E_) across all sites (19 sites, *N* = 304), ranging between 0.126 and 0.137 for *H*_O_ and 0.142 and 0.145 for *H*_E_. This corresponded with inbreeding coefficients that significantly differed across the sites (*F*_IS_ = 0.037–0.095; 95% CIs not overlapping zero; global *F*_IS_ = 0.07), which may indicate inbreeding or relatedness within sites, or it may be a result of underlying population substructure between sites (Table S5). Allelic richness (AR) ranged from 1.478 to 1.490 (Table S5), with no private alleles (PA) detected at any site. Global pairwise genetic differentiation among the sites was low, with an FST value of 0.001 (range −0.0009 to 0.0064) (Table S6). The highest differentiation was observed between two sites in regions 1 and 3. Genetic isolation by geographic distance was significant across sites (Mantel test, *r* = 0.31, *p* = 0.003; Figure S4a,b).

### 3.2 Genetic Structure

**FIGURE 2:**
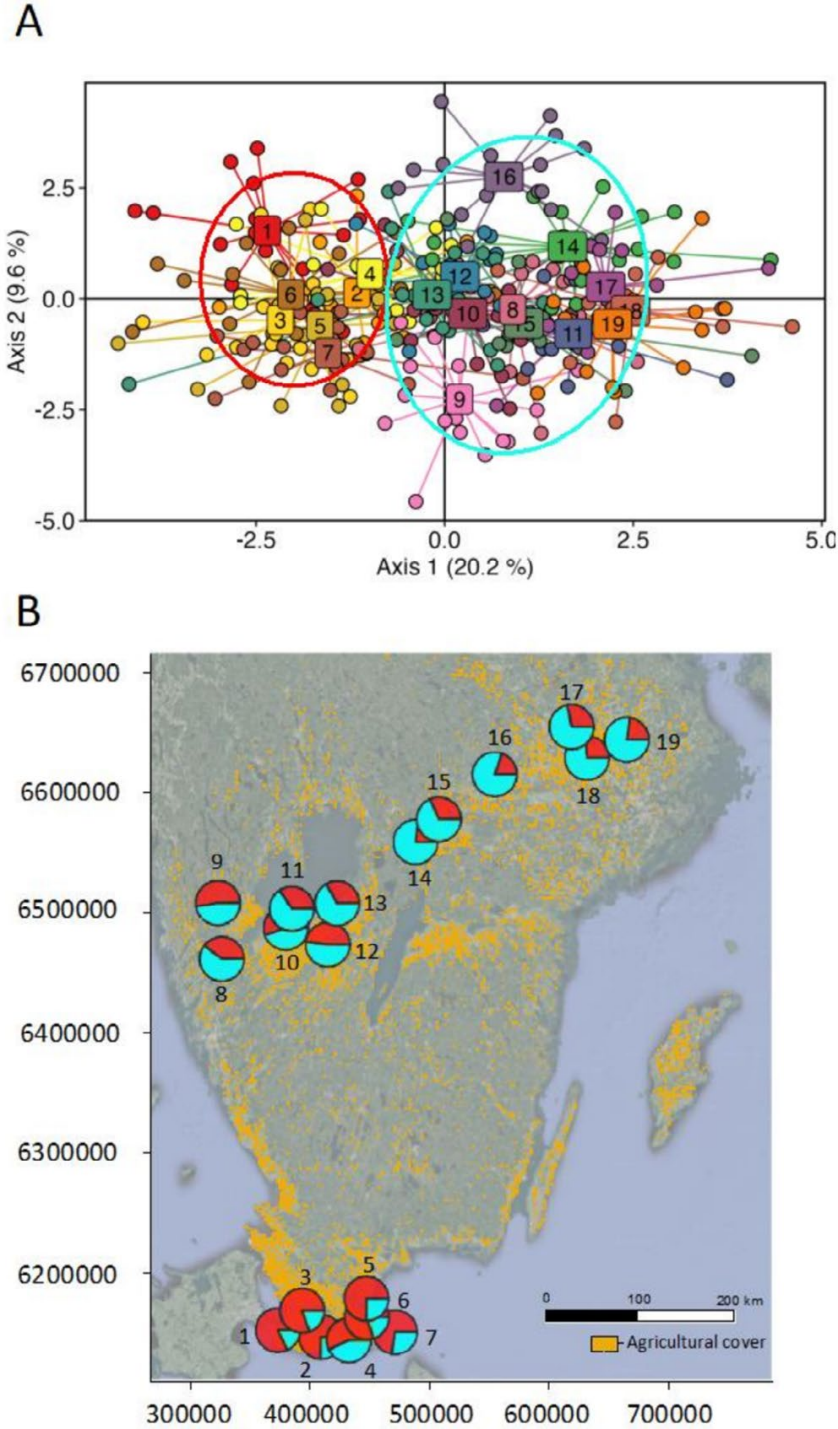
(A) Discriminant Analysis of Principal Components (DAPC), showing the genetic structure of *Bombus terrestris* from 19 sampling sites (*N* = 304 bees), separating region 1 (red circle) from regions 2 and 3 (blue circle); (B) genetic structure of *B. terrestris* displayed over agricultural cover with forest cover as the background. The proportion of red or blue at each site represents the mean assignment probability to either genetic cluster one or two (*K* = 2, admixture analysis).

The PCA revealed little genetic structure, with all 304 individuals clustering closely together (PCA axis 1: 0.7% of the variance) (Figure S5). The three separate PCAs of the samples from each region also formed one genetic cluster, although samples from region 3 were less tightly grouped compared to those in regions 1 and 2 (Figure S6a,c), suggesting more genetic differences among the samples in this region. The 19 sites showed some genetic clustering when using DAPC, with respect to latitude and region with sites in region 1 being those most separated from the sites in regions 2 and 3 (DAPC axis 1: 20.2% of the variance) (Figure 2A). The 10‐fold cross‐validation (CV) procedure of *K* = 1–5 in the ADMIXTURE analysis gave the highest support for one genetic cluster, *K* = 1 (0.317) (Figure S7), although *K* = 2 exhibited a similarly low CV error rate (0.323). Thus, we explored *K* = 2 and the sites in region 1 shared higher ancestry with each other than with the sites in regions 2 and 3, supporting *K* = 2 (Figure 2B; Figure S8). The co‐ancestry matrix generated by fineRADstructure showed little to no genetic divergence between the sites or regions (Figure S9). The clustering dendrogram formed 29 lineages with five clusters that were randomly found across the sampling distribution (Figure S9).

### 3.3 Effective Migration and Population Expansion

**FIGURE 3:**
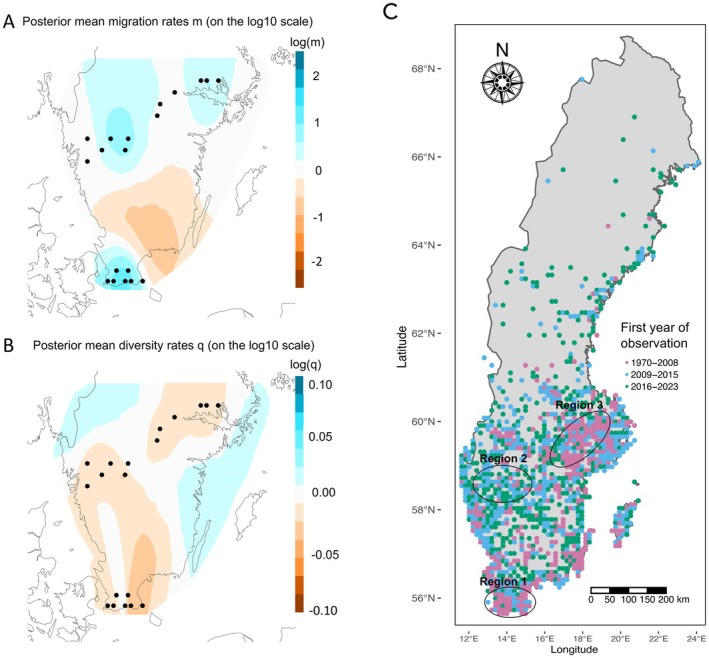
Surfaces of (A) effective migration (m) and (B) effective diversity (q) plots generated by EEMS of the Swedish *Bombus terrestris* population. Log(m) and log(*q*) = white represents average, blue represents higher‐than‐average, and brown represents lower‐than‐average effective migration and genetic diversity. The 19 sampling sites are represented as black circles; (C) map showing first‐year observation of *B. terrestris* across Sweden from 1970 to 2023 (GBIF). Pink dots represent the first observations between 1970 and 2008, blue dots between 2009 and 2015, and green dots between 2016 and 2023.

A negative global Tajima's *D* (−0.001) was found across all sites, as well as for each site independently (range between −0.262 and −0.308), indicating potential population expansion (Table S5). The estimated effective migration rate analysis (EEMS) revealed a region of lower‐than‐average effective migration, separating region 1 (south) from region 2 (central) and 3 (north) (Figure 3A). Two sites (3 and 4) in region 1 were located in areas with average effective genetic diversity estimated while all other sites in region 1 as well as in regions 2 and 3 were in areas with lower‐than‐average effective genetic diversity estimated (Figure 3B).

### 3.4 Environmental and Morphological Signatures of Selection

The univariate LFMM analysis did not detect any outlier SNPs associated with the tested landscape and climate sampling design or the other environmental and morphological predictors (GIF values ranged between 1.00 and 1.08). When considering the landscape design predictor variables using multivariate RDA, the full RDA model was significant (*p* = 0.001, df = 3, *F* = 1.1367; Table S7). The first axis was significant and explained 40% of the total variance and was used for outlier detection (Table S7). Using ±2.5 standard deviations as a cut‐off, 69 outlier SNPs were identified, of which 45 were associated with latitude and six with MaxTempWarmestMonth (Table 1; Figure S8). The full RDA model was also significant when running the environmental and morphological predictor variables (*p* = 0.001, df = 6, *F* = 1.0492; Table S9). Axis 1 was significant and explained 21% of the total variance in the RDA (Table S9). Using a ±2.5 standard deviation as a cut‐off, 76 outlier SNPs were identified of which 46 were associated with the percentage of agricultural land cover, three with MeanAnnualPrecip, 26 with percentage of urban cover, and one with body length (Table 1; Figure S10). In total 26 outlier SNPs overlapped between the two RDA models (Table S11).

**TABLE 1:** The candidate outliers SNPs (*N*) identified in the two separate RDA models for axis 1 for (A) the landscape and climate study design variables (e.g., those associated with ‘simple’ and ‘complex’ landscape and temperature) and (B), the other environmental and morphological predictor variables. The number of overlapping outlier SNPs between the two RDA analyses for RDA axis 1 is presented (see Table S11 for details).

	RDA1	Overlapping
(A) Landscape and climate study design variables	*N*	*N*
Latitude	45	25
MaxTempWarmestMonth	4	1
PC1_SNH_FB	0	—
(B) Other environmental and morphological variables		
% agricultural land cover	34	15
MeanAnnualPrecip	3	2
% urban land cover	13	8
Body length, mm	1	1
Total	101	26

### 3.5 Gene Annotations and Functions

The 145 outlier SNPs identified in the two RDA analyses mapped to 101 genes (landscape and climate design RDA1: 49 genes, Table S8, and environmental/morphology RDA1: 52 genes, Table S10) on the *B. terrestris* genome, of which only two genes were protein‐coding (missense and synonymous variant) and 20 genes possibly linked to protein‐coding genes, that were either 3′ or 5′ UTR variants (i.e., sequences at the beginning and end of a protein‐coding sequence) and upstream/downstream gene variants (Tables S8 and S10). The missense protein coding gene (transmembrane protein 132C) was found in the landscape and climate design RDA and associated with MaxTempWarmestMonth. The synonymous protein coding gene (odorant receptor 82a‐like) was found in both RDA analyses and associated with both latitude and percentage agricultural land cover (Tables S8 and S10). Three genes (glutamate receptor 1, acetylcholinesterase, and guanine nucleotide‐binding proteins) with possible protein‐coding effects were found in the environmental/morphology RDA and associated with the percentage of agricultural land cover (Table S10). In addition, other genes found with possible protein‐coding effects and associated with the percentage of agricultural land cover were cAMP‐specific 3′,5′‐cyclic phosphodiesterase, cadherin‐87A, and odorant receptor 82A. We also found genes with possible protein‐coding effects that were associated with latitude and the percentage of urban land cover; Mucin‐5AC protein and myocardin‐related transcription.

## 4 Discussion

We found little genetic structure and high gene flow in *B. terrestris* in Sweden, with some evidence for a northward range expansion and into more forested inland areas (Figures 2 and 3; Table S6). However, there was some support for genetic divergence between southern and northern regions (Figures 2 and Figure S4). This is potentially caused by a large, unsampled forested area between the regions where estimated effective migration rates were lower than average, acting as a possible dispersal barrier (Figure 3). Despite high gene flow, we identified signatures of local adaptation in *B. terrestris* as revealed by environmental association analyses and a multi‐variate ordination‐based outlier approach (Table 1; Figures S10 and S11). Several of the candidate loci mapped to genes involved in insecticide resistance (see section on Candidate genes under selection below), which may be an important trait for adaptation in areas with high agricultural cover. Overall, *B. terrestris* demonstrates high adaptive capacity under anthropogenic change, with its generalist traits enabling it to thrive in fragmented agricultural landscapes and persist under climate change and other environmental shifts. This is further supported by our evidence of its resilience, even in the absence of strong local adaptation and gene flow restrictions.

### 4.1 Low Genetic Diversity and Structure

The Swedish *B. terrestris* population exhibits lower observed genetic diversity (mean *H*_O_ = 0.132) across sampling sites compared to other European *B. terrestris* populations, such as across the Iberian Peninsula (per‐SNPs nucleotide diversity SNP (*π* ^SNP^), *H*_O_ = 0.2326) (Silva et al. 2020). Consistent with several studies of bumblebees (*Bombus* spp.) (SNPs WGS, Heraghty et al. 2023; SNPs RADsequencing, Jackson et al. 2018), we observed very low genetic differentiation between the study sites (Global *F*_ST_ = 0.001), and even lower in comparison with introduced populations of *B. terrestris* in Australia (*F*_ST_ = 0.005) (SNPs RADsequencing, Kardum Hjort, Paris, et al. 2023). These results demonstrate that the Swedish *B. terrestris* population is quite homogeneous with high gene flow between sub‐populations at different sampling sites and regions (i.e., regions 1–3).

Although the genetic structure was weak, two loosely clustered genetic groups were identified using DAPC and admixture analyses separated by southern and northern regions (Figure 2; Figure S8), also supported by significant isolation by distance (IBD) (Figure S4). The estimated effective genetic migration rate was also lower than average between these two groups (Figure 3A), which may be due to a large, forested area between the two regions that may act as a dispersal barrier between, limiting gene flow. Although bumblebees are generally considered highly dispersive (Fijen 2021), such landscape features, like large, forested regions, may still restrict movement and gene flow, leading to the formation of genetic clusters. Especially for *B. terrestris* , which typically inhabits areas with low‐density vegetation, such as agricultural landscapes, rather than dense forests (Geue and Thomassen 2020; Svensson et al. 2000). Although, *B. terrestris* is also known to occur in more densely vegetated areas (Svensson et al. 2000).

In the more northerly areas (approximately above 61° N latitude), the majority of the first observations of *B. terrestris* occurred within the last 7–14 years (2009–2023), with only occasional observations before 2009 (Figure 3C). In addition, observations of *B. terrestris* further inland and in more forested areas have been reported more recently (2009–2023) (Figure 2C). This suggests a recent population expansion of *B. terrestris* (cf. Herbertsson et al. 2021), which is consistent with the negative Tajima's *D* values we observed for each sampling site (Table S5). Such an expansion may be expected based on the climatic requirements of *B. terrestris* (Rasmont et al. 2015), including a general north‐northwest expansion previously documented (Martinet et al. 2015). The observed presence in more forested areas may reflect this broader geographic expansion, potentially facilitated by higher temperatures allowing the species to inhabit cooler environments, such as forests. This aligns with its classification as a broad habitat generalist, which may be more extensive than previously understood due to ongoing climate change.

### 4.2 Environmental Signatures of Selection

Our results showed limited genetic differentiation in the Swedish *B. terrestris* population, with only a modest number of outlier SNPs associated with environmental (*N* = 99) variables, and just 1 SNP with one morphological variable (Table 1). This lack of strong genetic structure is likely due to the extensive gene flow across the population, as well as the use of reduced representation sequencing, which only covers a small portion of the genome, possibly missing candidate genes under selection. Our results are consistent with other bumblebee studies, where another widespread generalist bumblebee, *Bombus vosnesenskii* , exhibited genetic adaptation in neuromuscular functions even with high gene flow and low population structure (global *F*_ST_ = 0.001) (Heraghty et al. 2023). Similarly, Jackson et al. (2020) found weak selection related to environmental and climatic variables in *B. vosnesenskii* .

Despite low genetic differentiation, signatures of selection related to urbanisation have been documented in *Bombus lapidarius* (Theodorou et al. 2018). A study by Hart et al. (2022) found a comparable number of candidate SNPs under high gene flow for two species, *B. lapidarius* and *B. pascuorum* , which were linked to agricultural stressors, including pesticide exposure. In contrast, stronger signals of selection in *B. terrestris* across Great Britain have been associated with neurological function and wing development (Colgan et al. 2022). Additionally, *B. terrestris* shows genetic adaptation in Tasmania, Australia, correlating with environmental conditions despite low genetic divergence (Kardum Hjort, Paris, et al. 2023; Kardum Hjort, Smith, et al. 2023). Together, these studies along with the present one demonstrate that local genetic adaptation can occur in bumblebees with high gene flow and minimal population structure. However, for the highly mobile *B. terrestris*, extensive gene flow appears to limit strong local genetic adaptation (Tigano and Friesen 2016; see also Kardum Hjort, Paris, et al. 2023). Furthermore, the generalist nature of *B. terrestris* and other wide‐ranging bumblebees (Hart et al. 2022; Heraghty et al. 2023; Svensson et al. 2000) may contribute to their lower sensitivity to anthropogenic change compared to specialist bees.

### 4.3 Candidate Genes Under Selection

Our candidate SNPs under selection were mostly associated with latitude (*N* = 45) and MaxTempWarmestMonth (*N* = 4) in the landscape and climate design RDA analysis (Table 1) and the percentage of agricultural land cover (*N* = 34) and percentage of urban land cover (*N* = 13) in the environmental and morphological RDA analysis (Table 1). However, only two genes had protein‐coding effects and 20 possible protein‐coding effects (Tables S8 and S10). We discuss the findings considering the gene functions found and their associations with environmental variables.

The signatures of selection we observed were not related to landscape complexity (i.e., PC1_SNH_FB) but rather to the total percentage of agricultural land cover. Several of the identified candidate outlier SNPs that are associated with the percentage of agricultural land cover are related to genes involved in insecticide resistance, which may be traits selected for in landscapes dominated by high agricultural land use (Tsvetkov et al. 2021). These outlier SNPs were related to genes involved in the glutamate receptor 1, acetylcholinesterase, and guanine nucleotide‐binding proteins. Previous studies on *Bombus* have found genes involved in detoxification to be under selection and were also associated with agricultural stressors, which may incorporate insecticide exposure (Hart et al. 2022). Notably, evidence for heritable genetic components of pesticide resistance have been found in honeybees (*Apis* sp.) (Tsvetkov et al. 2023), while several other insect species show adaptation to either agricultural environments or to insecticide/pesticide exposure (e.g., *Drosophila* sp. Menozzi et al. 2004, *Nilaparvata lugens* You et al. 2016, *Anopheles gambiae* Oliver and Brooke 2018, *Elateridae* Andrews et al. 2020).

Three other genes associated with variation in agricultural land cover (and latitude), cAMP‐specific 3′,5′‐cyclic phosphodiesterase, cadherin‐87A, and odorant receptor 82A, have been implicated in olfactory learning and memory (Davis et al. 1995) and infection response (Jiang et al. 2016). For social bees, olfaction and hygiene behaviour are often connected, where for example, olfactory cues are used to detect and subsequently remove parasites in honeybee colonies, which is essential for colony health and survival (Mondet et al. 2015).

We also documented outlier SNPs associated with latitude (and percentage of agricultural land cover) and the percentage of urban land cover that mapped to genes that are sensitive to insecticides, such as Mucin‐5AC protein (Li et al. 2022), and to genes (myocardin‐related transcription) involved in tracheal respiratory and wing development which might be related to urban stressors (Han et al. 2004). The function of one gene (transmembrane protein 132C) with protein‐coding effects and associated with MaxTempWarmestMonth is not known in *Bombus* nor as a homologue in *Drosophila melanogaster* .

Together these candidate genes may be under selection as a response to anthropogenic environmental change, particularly to the stressors of agricultural intensification. Twenty‐six outlier SNPs overlapped between the two RDA analyses and more precisely between latitude and the percentage of agricultural land cover, MaxTempWarmestMonth, or percentage of urban land cover (Table S11), therefore we cannot confidently tease apart which of these environmental variables is exerting a selection pressure. However, it is most likely that effects are exerted across more than one environmental or climatic variable, and several variables may interact to generate a selective response (González‐Varo et al. 2013). We further acknowledge that our observed selection signatures are not strong, with few candidate loci associated with agricultural stressors. Nevertheless, the potential role of these genes in how *B. terrestris* responds to anthropogenic environmental change via local adaptation is open for further validation.

## 5 Conclusions

We found that variation in landscape complexity has not resulted in genetic differences among *B. terrestris* populations in Sweden, as supported by a lack of selection signatures in response to the percentage of semi‐natural habitat and uncultivated agricultural field borders. These landscape variables may exert weak selection pressures on the bees, possibly because the species exhibits phenotypic plasticity or because these environmental features do not significantly influence bee fitness. Alternatively, the observed lack of differentiation could reflect homogenisation due to extensive gene flow, with populations being well‐mixed across the landscape. However, *B. terrestris* shows weak selective responses to another landscape variable, the percentage of agricultural land cover, including genes involved in insecticide resistance. As *B. terrestris* expands northwards and inland, its adaptive capacity seems to primarily operate at larger spatial scales, with limited evidence of local adaptation at smaller scales. This suggests that, while landscape simplification may drive broad‐scale adaptations, local genetic differentiation due to landscape features is more likely to occur under conditions of asymmetric dispersal or stronger selection pressures. While the range expansion of *B. terrestris* is likely influenced by broader environmental factors, such as climate change, this does not necessarily imply adaptation. The expansion may simply reflect a shift in the species' ecological niche without requiring genetic adaptation to new conditions. As such, the adaptive trajectory of *B. terrestris* may be more influenced by large‐scale processes, like climate change, rather than localised landscape or habitat changes.

## Author Contributions

C.K.H., J.E., P.O., R.Y.D. and H.G.S. conceptualised the study. C.K.H. collected the samples in the field. C.K.H. performed the statistical analyses. C.K.H. wrote the manuscript with editing and comments from all co‐authors.

## Conflicts of Interest

The authors declare no conflicts of interest.

## Supporting information


**Table S1:** The amount of semi‐natural habitat (SNH) (he).
**Table S2:** Summary table of samples included in the analyses (sample ID, N = 304).
**Table S3:** The three final predictors for the landscape and climate design analyses.
**Table S4:** Variance inflation factor (VIF) of the landscape and climate study design.
**Table S5:** Estimated allelic richness (A), expected heterozygosity (H), observed.
**Table S6:** Matrix representing bootstrapping (10,000 permutations) over loci of.
**Table S7:** Spearman rank correlations test of full time series and subset (2000–2003) time series.
**Table S8:** Full redundancy analysis (RDA) model for the landscape and climate sampling study predictors and significant axes used for outlier detection.
**Table S9:** Full redundancy analysis (RDA) model for the other environmental and morphological predictors and significant axes used for outlier detection.
**Table S10:** Overlapping outlier SNPs between the two RDA analyses for RDA axis 1.
**Table S11:** The 49 candidate outlier SNPs identified for RDA1 in the landscape and climate study design RDA analysis.
**Table S12:** The 52 candidate outlier SNPs identified for RDA1 in the other environmental and morphological RDA analysis.
**Figure S1:** (a) The percentage of semi‐natural habitat (SNH) and the percentage of agricultural cover, (b) the length of uncultivated field borders (km) and percentage of agricultural cover, and (c) the percentage of agricultural cover for the simple and complex study sites.
**Figure S2:** Pearson's correlation matrix for the landscape and climate study design predictor variables.
**Figure S3:** Pearson's correlation matrix for the other environmental and morphological predictor variables.
**Figure S4:** Isolation by Distance (IBD) analysis.
**Figure S5:** Principal component of Analysis (PCA) of the 304 B. terrestris individuals.
**Figure S6:** Principal component of Analysis (PCA) showing (a) region 1; (b) region 2.
**Figure S7:** Cross validation (CV) procedure of K = 1–5 in the ADMIXTURE.
**Figure S8:** The probability of ancestry to genetic clusters one (red) and two (blue).
**Figure S9:** A co‐ancestry matrix visualised as a heatmap, generated by fineRADstructure and RADpainter, showing little to nongenetic divergence among the samples.
**Figure S10:** Mean latitude (blue) and 90th percentile latitude (orange) of B. terrestris.
**Figure S11:** Candidate outlier SNPs (N = 69) identified on RDA axis 1 in the simple and complex landscape and temperature study design RDA analysis.
**Figure S12:** Candidate outlier SNPs (N = 76) identified on RDA axis 1 in the other environmental and morphological RDA analysis.

## Data Availability

The two final SNP datasets are available on Dryad https://doi.org/10.5061/dryad.f4qrfj76b.
