# Supplementary material for: Landscape and Climate‐Associated Selection in the Native and Widespread Bumblebee, Bombus terrestris
Source: Mol Ecol. 2025 Oct 9;34(21):e70141. doi: 10.1111/mec.70141 (PMC12573718; doi:10.1111/mec.70141)
Supplement: Supplementary file 1 — Table S1: The amount of semi‐natural habitat (SNH) (he). Table S2: Summary table of samples included in the analyses (sample ID, N = 304). Table S3: The three final predictors for the landscape and climate design analyses. Table S4: Variance inflation factor (VIF) of the landscape and climate study design. Table S5: Estimated allelic richness (A), expected heterozygosity (H), observed. Table S6: Matrix representing bootstrapping (10,000 permutations) over loci of. Table S7: Spearman rank correlations test of full time series and subset (2000–2003) time series. Table S8: Full redundancy analysis (RDA) model for the landscape and climate sampling study predictors and significant axes used for outlier detection. Table S9: Full redundancy analysis (RDA) model for the other environmental and morphological predictors and significant axes used for outlier detection. Table S10: Overlapping outlier SNPs between the two RDA analyses for RDA axis 1. Table S11: The 49 candidate outlier SNPs identified for RDA1 in the landscape and climate study design RDA analysis. Table S12: The 52 candidate outlier SNPs identified for RDA1 in the other environmental and morphological RDA analysis. Figure S1: (a) The percentage of semi‐natural habitat (SNH) and the percentage of agricultural cover, (b) the length of uncultivated field borders (km) and percentage of agricultural cover, and (c) the percentage of agricultural cover for the simple and complex study sites. Figure S2: Pearson's correlation matrix for the landscape and climate study design predictor variables. Figure S3: Pearson's correlation matrix for the other environmental and morphological predictor variables. Figure S4: Isolation by Distance (IBD) analysis. Figure S5: Principal component of Analysis (PCA) of the 304 B. terrestris individuals. Figure S6: Principal component of Analysis (PCA) showing (a) region 1; (b) region 2. Figure S7: Cross validation (CV) procedure of K = 1–5 in the ADMIXTURE. Figure S8: The probability of a [file MEC-34-e70141-s001.pdf]

Supplementary materials

Landscape and climate-associated selection in the native and widespread bumblebee, *Bombus terrestris*

Cecilia Kardum Hjort[1,2*], Rachael Y. Dudaniec[2], Peter Olsson[3], Johan Ekroos[3,4,5], Henrik G. Smith[1,3]

[1] Department of Biology, Lund University, SE-223 62 Lund, Sweden

[2] School of Natural Sciences, Macquarie University, Sydney, 2109 NSW, Australia

[3] Centre for Environmental and Climate Science, Lund University, SE-223 62 Lund, Sweden

[4] Department of Agricultural Sciences, Helsinki University, FI-00014, Finland

[5] Helsinki Institute of Sustainability Science, HELSUS, University of Helsinki, 00014 Helsinki, Finland

*Corresponding Author

Keywords

Bombus terrestris, landscape simplification, agriculture, selection, gene flow, neutral genetic divergence, morphology

Supplementary text

Text S1. Protocol for morphological measurements.

In the lab, each bumblebee was blotted on tissue paper and left to dry for 5 min at room temperature to allow ethanol to evaporate, and then weighed (0.001 g precision) with a fine scale. Inter-tegular distance (ITD, 0.01 mm precision) was measured for each bumblebee using digital callipers. Bumblebees were placed ventrally on millimetre paper and photographed using a digital camera microscope (Veho DX-2 USB 5MP Microscope). Subsequently, each bumblebee had one wing-pair (i.e., the most intact small and large wing, respectively) carefully dissected and placed on millimetre-paper and photographed under the camera microscope. Body length (mm), wing length (mm), and wing area (mm^2) measurements were measured from photographic images at 0.01 mm precision using ImageJ v1.5.3 (Schneider et al. 2012). To characterize wing morphology while helping to control for body size variation among *B. terrestris* workers, we calculated wing loading as the body weight in grams divided by the total wing area in cm^2.

Text S2. Purification of DNA from *B. terrestris* for COI barcoding using a HotSHOT Protocol.

For each sample, break the femur (the tibia or tarsus also works) off from the leg using a tweezer. Put the segment in the new tube labelled with the corresponding sample name and break it apart with the tip of the tweezer. Make sure that as few segments as possible sticks to the tweezer. Put the used tweezer in a beaker with 70 % Ethanol. (Remember to work under the fume hood, wear lab coat and latex gloves when working with acetone).

Put the old tubes back in the freezer and leave the new tubes open to dry under the fume hood overnight or use the vacuum centrifuge (If you plan to use the vacuum centrifuge, turn on the cooling trap 1 hour before this step).

Wash the tips of the tweezers with dH_2O and dry them a bit with paper. Burn the tweezers in the Bunsen burner. Hold the end of the tweezer and burn as much of it as possible. Finish by holding the tips in the flame of the Bunsen burner until they are weakly glowing red, then place them with the tip downwards in a clean beaker. When they have cooled, put them back in the box.

Turn the Thermo-Shaker on. Set the temperature at 95 ºC and without any shaking. Nitrile gloves are needed from step 6 to step 9.

Add 75 µl of Alkaline Lysis Reagent (25 mM NaOH, 0.2 mM EDTA, pH=12) to each tube (remember to make a blank for the NanoDrop). Close the tubes, then mix and spin the samples briefly.

Heat the samples at 95 ºC for 15-20 minutes without any shaking in the Thermo-Shaker. Prepare a bowl of ice and 1.5 ml screw cork Cryo tubes for step 11, labelled with the name of each sample.

Remove the tubes from the Thermo-Shaker and spin them briefly to remove the condensation on the lid. Place the tubes on ice with the lid a bit above the ice to cool the tubes down and to avoid new condensation on the lid.

When the samples have been cooled, add 75 µl of the neutralizing reagent (40 mM Tris-HCl, pH=5). Close the tubes, then mix and spin the samples.

Spin the samples at top speed for a short pulse to move the fragments to the bottom of the tubes.

Remove 100 µl of the liquid without disturbing the fragments and put it in a new tube labelled with the corresponding sample name. If a sample is disturbed, spin it and try again.

Mix the samples, then quantify the samples with the NanoDrop. Blank with the solution made during the extraction. Make sure that the drop touches both the bottom and the top of the scanner when it separates (this solution sometimes split during scanning). If it splits, first try scanning the sample again without changing the drop. Store the samples at -20 ºC or colder.

Text S3. COI mitochondrial gene amplification following Wahlberg and Wheat (2008).

The COI mitochondrial gene was amplified in all samples using the universal primer-pair:

HybLCO 5' TAA TAC GAC TCA CTA TAG GGG GTC AAC AAA TCA TAA AGA TAT

TGG 3' and HybHCO 5' ATT AAC CCT CAC TAA AGG GT AAA CTT CAG GGT GAC

CAA AAA ATC A 3'. The PCR cycling conditions were as follows: 95°C for 5 min, 40 cycles of 94°C for 30 sec, 50°C for 30 sec and 72°C for 1 min 30 sec, with a final extension period of 72°C for 10 min. The PCR product was purified using 0.9 µl of ExoI (ThermoFisher Scientific) accordingly to Wahlberg & Wheat (2008) and submitted to Macrogen Europe (https://dna.macrogen-europe.com/) for Sanger sequencing.

Text S4. Protocol for DNA extractions performed by DarT

DNA extraction was performed using the automated DNA extraction protocol on a 'TECAN freedom evo 100' liquid handling robot with a commercially available magnetic bead-based DNA extraction kit (Macherey-Nagel). DNA digestion and ligation reactions were performed as per Kilian et al. (2012). Digested and ligated samples were amplified for 30 cycles using the following PCR conditions: 94°C for 1 min, 94°C for 20 sec, 58°C for 30 sec, 72°C for 45 sec, 72°C for 7 min.

Text S5. DArT SNP calling, filtering and quality check

Proprietary analytical pipelines (DArTsoft14 – DArT's proprietary software) were used to process sequences generated from each lane. DArT uses a reproducibility score (i.e. the proportion of technical replicate assay pairs for which the marker score is consistent) and polymorphism information content (PIC: an index ranging from zero to one and inform allele variations of SNP marker) to assess the quality and information content of SNP calls. The threshold for the reproducibility score is set to 97%. Further quality control was undertaken by removing any bacterial or viral contaminant sequences using alignment to matching sequences within the GenBank and DArT databases.

Supplementary tables

Table S1. The amount of semi-natural habitat (SNH) (he), uncultivated agricultural field borders (km), and maximum temperature of the warmest month (MaxTempWarmestMonth, °C) for simple and complex sampling locations within regions 1, 2, and 3.

	Simple			Complex		
	SNH (ha)	Field borders (km)	MaxTemp Warmest Month (°C)	SNH (ha)	Field borders (km)	MaxTemp Warmest Month (°C)
**Region 1**	10-16	220-260	20.89-21.16	131-180	280-450	20.52-21.14
**Region 2**	10-19	190-230	20.73-21.26	132-195	300-360	20.55-21.36
**Region 3**	12-19	200-230	22.08-22.18	129-189	280-350	21.67-22.20

Table S2. Summary table of samples included in the analyses (sample ID, N=304), corresponding site ID, longitude and latitude, landscape type (i.e. simple or complex), region, climate pair based on MaxTempWarmestMonth (°C), MeanAnnualTemp (°C), MeanAnnualPrecip (mm), SeasonPrecip (%), AvgSummerWind (m/s), Canopy height (m), Percentage SNH, uncultivated agricultural field borders (km), percentage agricultural land cover, percentage urban cover, percentage forest cover, ITD (mm), body weight (g), body length (mm), small and large wing length (mm), small and large wing area (mm^2), wing pair total area (mm^2) and wing loading (g/cm^2).

Sample ID	Site ID map	X	Y	Landscape type	Region	Climate pair	MaxTemp WarmestMonth	MeanAnnualTemp	MeanAnnual Precip	SeasonPrecip	AvgSummerWind	Canopy height
460_1_SJE_10	1	13.005262	55.4934613	S	1	5	20.94	8.14	555.98	18.80	4.18	2.16
460_1_CH_16	1	13.005262	55.4934613	S	1	5	20.94	8.14	555.98	18.80	4.18	2.16
460_1_CH_30	1	13.005262	55.4934613	S	1	5	20.94	8.14	555.98	18.80	4.18	2.16
460_1_SJE_01	1	13.005262	55.4934613	S	1	5	20.94	8.14	555.98	18.80	4.18	2.16
460_1_CH_02	1	13.005262	55.4934613	S	1	5	20.94	8.14	555.98	18.80	4.18	2.16
460_2_MV_14	1	13.005262	55.4934613	S	1	5	20.94	8.14	555.98	18.80	4.18	2.16
460_2_SJE_25	1	13.005262	55.4934613	S	1	5	20.94	8.14	555.98	18.80	4.18	2.16
460_1_CH_08	1	13.005262	55.4934613	S	1	5	20.94	8.14	555.98	18.80	4.18	2.16
460_2_SJE_19	1	13.005262	55.4934613	S	1	5	20.94	8.14	555.98	18.80	4.18	2.16
460_2_SJE_21	1	13.005262	55.4934613	S	1	5	20.94	8.14	555.98	18.80	4.18	2.16
460_2_SJE_09	1	13.005262	55.4934613	S	1	5	20.94	8.14	555.98	18.80	4.18	2.16
460_1_SJE_13	1	13.005262	55.4934613	S	1	5	20.94	8.14	555.98	18.80	4.18	2.16
460_1_SJE_16	1	13.005262	55.4934613	S	1	5	20.94	8.14	555.98	18.80	4.18	2.16
460_2_SJE_08	1	13.005262	55.4934613	S	1	5	20.94	8.14	555.98	18.80	4.18	2.16
460_1_SJE_06	1	13.005262	55.4934613	S	1	5	20.94	8.14	555.98	18.80	4.18	2.16
460_2_SJE_27	1	13.005262	55.4934613	S	1	5	20.94	8.14	555.98	18.80	4.18	2.16
784_1_SJE_12	2	13.292356	55.443879	S	1	1	20.90	7.79	670.34	21.31	3.99	2.45
784_1_CH_02	2	13.292356	55.443879	S	1	1	20.90	7.79	670.34	21.31	3.99	2.45
784_1_CH_11X	2	13.292356	55.443879	S	1	1	20.90	7.79	670.34	21.31	3.99	2.45
784_1_SJE_22	2	13.292356	55.443879	S	1	1	20.90	7.79	670.34	21.31	3.99	2.45
784_1_CH_09	2	13.292356	55.443879	S	1	1	20.90	7.79	670.34	21.31	3.99	2.45
784_2_SJE_15	2	13.292356	55.443879	S	1	1	20.90	7.79	670.34	21.31	3.99	2.45
784_2_MV_18	2	13.292356	55.443879	S	1	1	20.90	7.79	670.34	21.31	3.99	2.45
784_1_SJE_07	2	13.292356	55.443879	S	1	1	20.90	7.79	670.34	21.31	3.99	2.45
784_2_SJE_14	2	13.292356	55.443879	S	1	1	20.90	7.79	670.34	21.31	3.99	2.45
784_1_CH_9X	2	13.292356	55.443879	S	1	1	20.90	7.79	670.34	21.31	3.99	2.45
784_1_SJE_21	2	13.292356	55.443879	S	1	1	20.90	7.79	670.34	21.31	3.99	2.45
784_2_SJE_21	2	13.292356	55.443879	S	1	1	20.90	7.79	670.34	21.31	3.99	2.45
784_2_MV_03	2	13.292356	55.443879	S	1	1	20.90	7.79	670.34	21.31	3.99	2.45
784_2_SJE_18	2	13.292356	55.443879	S	1	1	20.90	7.79	670.34	21.31	3.99	2.45
784_2_SJE_01	2	13.292356	55.443879	S	1	1	20.90	7.79	670.34	21.31	3.99	2.45
784_2_SJE_07	2	13.292356	55.443879	S	1	1	20.90	7.79	670.34	21.31	3.99	2.45
839_1_CH_23	3	13.3317906	55.6331503	S	1	6	21.16	7.96	657.66	21.48	3.16	1.91
839_1_SJE_05	3	13.3317906	55.6331503	S	1	6	21.16	7.96	657.66	21.48	3.16	1.91
839_1_CH_02	3	13.3317906	55.6331503	S	1	6	21.16	7.96	657.66	21.48	3.16	1.91
839_1_SJE_06	3	13.3317906	55.6331503	S	1	6	21.16	7.96	657.66	21.48	3.16	1.91
839_1_SJE_1ter	3	13.3317906	55.6331503	S	1	6	21.16	7.96	657.66	21.48	3.16	1.91
839_2_CH_04	3	13.3317906	55.6331503	S	1	6	21.16	7.96	657.66	21.48	3.16	1.91

839_2_CH_02	3	13.3317906	55.6331503	S	1	6	21.16	7.96	657.66	21.48	3.16	1.91
839_1_CH_12	3	13.3317906	55.6331503	S	1	6	21.16	7.96	657.66	21.48	3.16	1.91
839_2_CH_06	3	13.3317906	55.6331503	S	1	6	21.16	7.96	657.66	21.48	3.16	1.91
839_1_CH_21	3	13.3317906	55.6331503	S	1	6	21.16	7.96	657.66	21.48	3.16	1.91
839_2_CH_22	3	13.3317906	55.6331503	S	1	6	21.16	7.96	657.66	21.48	3.16	1.91
839_1_SJE_2ter	3	13.3317906	55.6331503	S	1	6	21.16	7.96	657.66	21.48	3.16	1.91
839_1_SJE_01	3	13.3317906	55.6331503	S	1	6	21.16	7.96	657.66	21.48	3.16	1.91
839_1_SJE_16	3	13.3317906	55.6331503	S	1	6	21.16	7.96	657.66	21.48	3.16	1.91
839_1_CH_14	3	13.3317906	55.6331503	S	1	6	21.16	7.96	657.66	21.48	3.16	1.91
839_2_CH_17	3	13.3317906	55.6331503	S	1	6	21.16	7.96	657.66	21.48	3.16	1.91
839_1_CH_5ter	3	13.3317906	55.6331503	S	1	6	21.16	7.96	657.66	21.48	3.16	1.91
1192_2_CH_19	4	13.8130747	55.4769976	C	1	4	21.14	7.74	663.38	20.88	4.23	2.89
1192_1_CH_04	4	13.8130747	55.4769976	C	1	4	21.14	7.74	663.38	20.88	4.23	2.89
1192_2_CH_10	4	13.8130747	55.4769976	C	1	4	21.14	7.74	663.38	20.88	4.23	2.89
1192_2_CH_32	4	13.8130747	55.4769976	C	1	4	21.14	7.74	663.38	20.88	4.23	2.89
1192_1_SJE_30	4	13.8130747	55.4769976	C	1	4	21.14	7.74	663.38	20.88	4.23	2.89
1192_1_CH_11X	4	13.8130747	55.4769976	C	1	4	21.14	7.74	663.38	20.88	4.23	2.89
1192_2_CH_14	4	13.8130747	55.4769976	C	1	4	21.14	7.74	663.38	20.88	4.23	2.89
1192_2_CH_31	4	13.8130747	55.4769976	C	1	4	21.14	7.74	663.38	20.88	4.23	2.89
1192_1_CH_1X	4	13.8130747	55.4769976	C	1	4	21.14	7.74	663.38	20.88	4.23	2.89
1192_1_SJE_20	4	13.8130747	55.4769976	C	1	4	21.14	7.74	663.38	20.88	4.23	2.89
1192_2_CH_04	4	13.8130747	55.4769976	C	1	4	21.14	7.74	663.38	20.88	4.23	2.89
1192_1_SJE_03	4	13.8130747	55.4769976	C	1	4	21.14	7.74	663.38	20.88	4.23	2.89
1192_2_CH_21	4	13.8130747	55.4769976	C	1	4	21.14	7.74	663.38	20.88	4.23	2.89
1192_1_CH_14	4	13.8130747	55.4769976	C	1	4	21.14	7.74	663.38	20.88	4.23	2.89
1192_2_CH_20	4	13.8130747	55.4769976	C	1	4	21.14	7.74	663.38	20.88	4.23	2.89
1192_1_SJE_27	4	13.8130747	55.4769976	C	1	4	21.14	7.74	663.38	20.88	4.23	2.89
1192_1_SJE_05	4	13.8130747	55.4769976	C	1	4	21.14	7.74	663.38	20.88	4.23	2.89
1265_1_SJE_14	5	13.9012236	55.720446	C	1	5	20.52	6.99	787.44	20.65	3.38	8.24
1265_1_SJE_21	5	13.9012236	55.720446	C	1	5	20.52	6.99	787.44	20.65	3.38	8.24
1265_2_CH_21	5	13.9012236	55.720446	C	1	5	20.52	6.99	787.44	20.65	3.38	8.24
1265_1_SJE_18	5	13.9012236	55.720446	C	1	5	20.52	6.99	787.44	20.65	3.38	8.24
1265_2_CH_05	5	13.9012236	55.720446	C	1	5	20.52	6.99	787.44	20.65	3.38	8.24
1265_2_CH_37	5	13.9012236	55.720446	C	1	5	20.52	6.99	787.44	20.65	3.38	8.24
1265_1_CH_06	5	13.9012236	55.720446	C	1	5	20.52	6.99	787.44	20.65	3.38	8.24
1265_2_CH_15	5	13.9012236	55.720446	C	1	5	20.52	6.99	787.44	20.65	3.38	8.24
1265_2_CH_20	5	13.9012236	55.720446	C	1	5	20.52	6.99	787.44	20.65	3.38	8.24
1265_2_CH_36	5	13.9012236	55.720446	C	1	5	20.52	6.99	787.44	20.65	3.38	8.24
1265_2_CH_17	5	13.9012236	55.720446	C	1	5	20.52	6.99	787.44	20.65	3.38	8.24
1265_1_CH_01	5	13.9012236	55.720446	C	1	5	20.52	6.99	787.44	20.65	3.38	8.24
1265_1_SJE_22	5	13.9012236	55.720446	C	1	5	20.52	6.99	787.44	20.65	3.38	8.24
1265_1_SJE_20	5	13.9012236	55.720446	C	1	5	20.52	6.99	787.44	20.65	3.38	8.24
1265_2_CH_09	5	13.9012236	55.720446	C	1	5	20.52	6.99	787.44	20.65	3.38	8.24
1265_1_CH_23	5	13.9012236	55.720446	C	1	5	20.52	6.99	787.44	20.65	3.38	8.24
1265_1_SJE_13	5	13.9012236	55.720446	C	1	5	20.52	6.99	787.44	20.65	3.38	8.24
1326_1_CH_31	6	13.9049913	55.5856889	C	1	3	21.13	7.51	697.72	20.77	3.78	7.62
1326_2_CH_50	6	13.9049913	55.5856889	C	1	3	21.13	7.51	697.72	20.77	3.78	7.62
1326_2_CH_42	6	13.9049913	55.5856889	C	1	3	21.13	7.51	697.72	20.77	3.78	7.62
1326_2_CH_35	6	13.9049913	55.5856889	C	1	3	21.13	7.51	697.72	20.77	3.78	7.62
1326_1_CH_05	6	13.9049913	55.5856889	C	1	3	21.13	7.51	697.72	20.77	3.78	7.62
1326_1_SJE_04	6	13.9049913	55.5856889	C	1	3	21.13	7.51	697.72	20.77	3.78	7.62
1326_2_CH_20	6	13.9049913	55.5856889	C	1	3	21.13	7.51	697.72	20.77	3.78	7.62
1326_1_SJE_03	6	13.9049913	55.5856889	C	1	3	21.13	7.51	697.72	20.77	3.78	7.62
1326_2_CH_04	6	13.9049913	55.5856889	C	1	3	21.13	7.51	697.72	20.77	3.78	7.62

1326_2_CH_07	6	13.9049913	55.5856889	C	1	3	21.13	7.51	697.72	20.77	3.78	7.62
1326_1_CH_30	6	13.9049913	55.5856889	C	1	3	21.13	7.51	697.72	20.77	3.78	7.62
1326_1_SJE_14	6	13.9049913	55.5856889	C	1	3	21.13	7.51	697.72	20.77	3.78	7.62
1326_2_CH_37	6	13.9049913	55.5856889	C	1	3	21.13	7.51	697.72	20.77	3.78	7.62
1326_1_CH_07	6	13.9049913	55.5856889	C	1	3	21.13	7.51	697.72	20.77	3.78	7.62
1326_2_CH_32	6	13.9049913	55.5856889	C	1	3	21.13	7.51	697.72	20.77	3.78	7.62
1326_1_SJE_02	6	13.9049913	55.5856889	C	1	3	21.13	7.51	697.72	20.77	3.78	7.62
1326_1_CH_26	6	13.9049913	55.5856889	C	1	3	21.13	7.51	697.72	20.77	3.78	7.62
1525_1_CH_20	7	14.2866857	55.5345885	C	1	1	20.85	7.84	635.49	19.83	4.31	2.89
1525_1_CH_22	7	14.2866857	55.5345885	C	1	1	20.85	7.84	635.49	19.83	4.31	2.89
1525_2_CH_02	7	14.2866857	55.5345885	C	1	1	20.85	7.84	635.49	19.83	4.31	2.89
1525_2_CH_21	7	14.2866857	55.5345885	C	1	1	20.85	7.84	635.49	19.83	4.31	2.89
1525_1_CH_15	7	14.2866857	55.5345885	C	1	1	20.85	7.84	635.49	19.83	4.31	2.89
1525_1_SJE_01	7	14.2866857	55.5345885	C	1	1	20.85	7.84	635.49	19.83	4.31	2.89
1525_2_CH_19	7	14.2866857	55.5345885	C	1	1	20.85	7.84	635.49	19.83	4.31	2.89
1525_2_CH_15	7	14.2866857	55.5345885	C	1	1	20.85	7.84	635.49	19.83	4.31	2.89
1525_1_CH_12	7	14.2866857	55.5345885	C	1	1	20.85	7.84	635.49	19.83	4.31	2.89
1525_1_CH_17	7	14.2866857	55.5345885	C	1	1	20.85	7.84	635.49	19.83	4.31	2.89
1525_1_SJE_17	7	14.2866857	55.5345885	C	1	1	20.85	7.84	635.49	19.83	4.31	2.89
1525_2_CH_32	7	14.2866857	55.5345885	C	1	1	20.85	7.84	635.49	19.83	4.31	2.89
1525_1_CH_06	7	14.2866857	55.5345885	C	1	1	20.85	7.84	635.49	19.83	4.31	2.89
1525_2_CH_18	7	14.2866857	55.5345885	C	1	1	20.85	7.84	635.49	19.83	4.31	2.89
1525_1_SJE_15	7	14.2866857	55.5345885	C	1	1	20.85	7.84	635.49	19.83	4.31	2.89
1525_2_CH_10	7	14.2866857	55.5345885	C	1	1	20.85	7.84	635.49	19.83	4.31	2.89
1525_2_CH_16	7	14.2866857	55.5345885	C	1	1	20.85	7.84	635.49	19.83	4.31	2.89
2387_1_SJE_10	8	12.2519977	58.1674249	C	4	7	20.55	6.63	797.56	22.11	3.17	8.79
2387_1_SJE_01	8	12.2519977	58.1674249	C	4	7	20.55	6.63	797.56	22.11	3.17	8.79
2387_2_SJE_04	8	12.2519977	58.1674249	C	4	7	20.55	6.63	797.56	22.11	3.17	8.79
2387_1_SJE_24	8	12.2519977	58.1674249	C	4	7	20.55	6.63	797.56	22.11	3.17	8.79
2387_2_SJE_24	8	12.2519977	58.1674249	C	4	7	20.55	6.63	797.56	22.11	3.17	8.79
2387_2_SJE_10	8	12.2519977	58.1674249	C	4	7	20.55	6.63	797.56	22.11	3.17	8.79
2387_2_SJE_11	8	12.2519977	58.1674249	C	4	7	20.55	6.63	797.56	22.11	3.17	8.79
2387_1_SJE_16	8	12.2519977	58.1674249	C	4	7	20.55	6.63	797.56	22.11	3.17	8.79
2387_1_SJE_29	8	12.2519977	58.1674249	C	4	7	20.55	6.63	797.56	22.11	3.17	8.79
2387_1_SJE_12	8	12.2519977	58.1674249	C	4	7	20.55	6.63	797.56	22.11	3.17	8.79
2387_1_SJE_14	8	12.2519977	58.1674249	C	4	7	20.55	6.63	797.56	22.11	3.17	8.79
2387_1_SJE_17	8	12.2519977	58.1674249	C	4	7	20.55	6.63	797.56	22.11	3.17	8.79
2387_2_SJE_06	8	12.2519977	58.1674249	C	4	7	20.55	6.63	797.56	22.11	3.17	8.79
2387_2_SJE_27	8	12.2519977	58.1674249	C	4	7	20.55	6.63	797.56	22.11	3.17	8.79
2387_1_SJE_25	8	12.2519977	58.1674249	C	4	7	20.55	6.63	797.56	22.11	3.17	8.79
2387_1_SJE_30	8	12.2519977	58.1674249	C	4	7	20.55	6.63	797.56	22.11	3.17	8.79
2387_1_SJE_11	8	12.2519977	58.1674249	C	4	7	20.55	6.63	797.56	22.11	3.17	8.79
2402_2_SJE_32	9	12.2204161	58.5711235	S	4	7	20.73	6.26	810.28	23.99	2.98	6.41
2402_2_SJE_29	9	12.2204161	58.5711235	S	4	7	20.73	6.26	810.28	23.99	2.98	6.41
2402_1_SJE_29	9	12.2204161	58.5711235	S	4	7	20.73	6.26	810.28	23.99	2.98	6.41
2402_2_SJE_04	9	12.2204161	58.5711235	S	4	7	20.73	6.26	810.28	23.99	2.98	6.41
2402_2_SJE_18	9	12.2204161	58.5711235	S	4	7	20.73	6.26	810.28	23.99	2.98	6.41
2402_1_SJE_33	9	12.2204161	58.5711235	S	4	7	20.73	6.26	810.28	23.99	2.98	6.41
2402_1_SJE_08	9	12.2204161	58.5711235	S	4	7	20.73	6.26	810.28	23.99	2.98	6.41
2402_1_SJE_34	9	12.2204161	58.5711235	S	4	7	20.73	6.26	810.28	23.99	2.98	6.41
2402_2_SJE_1ter	9	12.2204161	58.5711235	S	4	7	20.73	6.26	810.28	23.99	2.98	6.41
2402_2_SJE_12	9	12.2204161	58.5711235	S	4	7	20.73	6.26	810.28	23.99	2.98	6.41
2402_1_SJE_28	9	12.2204161	58.5711235	S	4	7	20.73	6.26	810.28	23.99	2.98	6.41
2402_1_SJE_06	9	12.2204161	58.5711235	S	4	7	20.73	6.26	810.28	23.99	2.98	6.41

2402_2_SJE_4ter	9	12.2204161	58.5711235	S	4	7	20.73	6.26	810.28	23.99	2.98	6.41
2402_2_SJE_34	9	12.2204161	58.5711235	S	4	7	20.73	6.26	810.28	23.99	2.98	6.41
2402_1_SJE_32	9	12.2204161	58.5711235	S	4	7	20.73	6.26	810.28	23.99	2.98	6.41
2402_2_SJE_03	9	12.2204161	58.5711235	S	4	7	20.73	6.26	810.28	23.99	2.98	6.41
2732_1_SJE_09	10	12.795779	58.4475187	S	4	8	21.11	6.60	626.41	26.48	2.97	8.05
2732_2_SJE_37	10	12.795779	58.4475187	S	4	8	21.11	6.60	626.41	26.48	2.97	8.05
2732_1_SJE_29	10	12.795779	58.4475187	S	4	8	21.11	6.60	626.41	26.48	2.97	8.05
2732_2_SJE_06	10	12.795779	58.4475187	S	4	8	21.11	6.60	626.41	26.48	2.97	8.05
2732_1_SJE_16	10	12.795779	58.4475187	S	4	8	21.11	6.60	626.41	26.48	2.97	8.05
2732_2_SJE_05	10	12.795779	58.4475187	S	4	8	21.11	6.60	626.41	26.48	2.97	8.05
2732_1_SJE_17	10	12.795779	58.4475187	S	4	8	21.11	6.60	626.41	26.48	2.97	8.05
2732_1_SJE_20	10	12.795779	58.4475187	S	4	8	21.11	6.60	626.41	26.48	2.97	8.05
2732_1_SJE_28	10	12.795779	58.4475187	S	4	8	21.11	6.60	626.41	26.48	2.97	8.05
2732_2_SJE_18	10	12.795779	58.4475187	S	4	8	21.11	6.60	626.41	26.48	2.97	8.05
2732_2_SJE_39	10	12.795779	58.4475187	S	4	8	21.11	6.60	626.41	26.48	2.97	8.05
2732_1_SJE_15	10	12.795779	58.4475187	S	4	8	21.11	6.60	626.41	26.48	2.97	8.05
2732_1_SJE_08	10	12.795779	58.4475187	S	4	8	21.11	6.60	626.41	26.48	2.97	8.05
2732_1_SJE_30	10	12.795779	58.4475187	S	4	8	21.11	6.60	626.41	26.48	2.97	8.05
2732_2_SJE_44	10	12.795779	58.4475187	S	4	8	21.11	6.60	626.41	26.48	2.97	8.05
2732_2_SJE_21	10	12.795779	58.4475187	S	4	8	21.11	6.60	626.41	26.48	2.97	8.05
2732_2_SJE_42	10	12.795779	58.4475187	S	4	8	21.11	6.60	626.41	26.48	2.97	8.05
2844_1_SJE_33	11	13.0451303	58.5863189	C	4	8	21.38	6.57	623.43	25.96	3.15	10.12
2844_1_SJE_12	11	13.0451303	58.5863189	C	4	8	21.38	6.57	623.43	25.96	3.15	10.12
2844_2_SJE_20	11	13.0451303	58.5863189	C	4	8	21.38	6.57	623.43	25.96	3.15	10.12
2844_2_SJE_10	11	13.0451303	58.5863189	C	4	8	21.38	6.57	623.43	25.96	3.15	10.12
2844_1_SJE_19	11	13.0451303	58.5863189	C	4	8	21.38	6.57	623.43	25.96	3.15	10.12
2844_2_SJE_27	11	13.0451303	58.5863189	C	4	8	21.38	6.57	623.43	25.96	3.15	10.12
2844_2_SJE_4ter	11	13.0451303	58.5863189	C	4	8	21.38	6.57	623.43	25.96	3.15	10.12
2844_2_SJE_14	11	13.0451303	58.5863189	C	4	8	21.38	6.57	623.43	25.96	3.15	10.12
2844_2_SJE_22	11	13.0451303	58.5863189	C	4	8	21.38	6.57	623.43	25.96	3.15	10.12
2844_1_SJE_11	11	13.0451303	58.5863189	C	4	8	21.38	6.57	623.43	25.96	3.15	10.12
2844_2_SJE_1ter	11	13.0451303	58.5863189	C	4	8	21.38	6.57	623.43	25.96	3.15	10.12
2844_2_SJE_15	11	13.0451303	58.5863189	C	4	8	21.38	6.57	623.43	25.96	3.15	10.12
2844_2_SJE_36	11	13.0451303	58.5863189	C	4	8	21.38	6.57	623.43	25.96	3.15	10.12
2844_1_SJE_1ter	11	13.0451303	58.5863189	C	4	8	21.38	6.57	623.43	25.96	3.15	10.12
3172_2_SJE_19	12	13.6763319	58.2709974	C	4	9	20.65	5.86	671.58	21.85	3.33	6.84
3172_1_SJE_32	12	13.6763319	58.2709974	C	4	9	20.65	5.86	671.58	21.85	3.33	6.84
3172_1_SJE_41	12	13.6763319	58.2709974	C	4	9	20.65	5.86	671.58	21.85	3.33	6.84
3172_2_SJE_02	12	13.6763319	58.2709974	C	4	9	20.65	5.86	671.58	21.85	3.33	6.84
3172_1_SJE_39	12	13.6763319	58.2709974	C	4	9	20.65	5.86	671.58	21.85	3.33	6.84
3172_2_SJE_39	12	13.6763319	58.2709974	C	4	9	20.65	5.86	671.58	21.85	3.33	6.84
3172_2_SJE_40	12	13.6763319	58.2709974	C	4	9	20.65	5.86	671.58	21.85	3.33	6.84
3172_1_SJE_05	12	13.6763319	58.2709974	C	4	9	20.65	5.86	671.58	21.85	3.33	6.84
3172_2_SJE_20	12	13.6763319	58.2709974	C	4	9	20.65	5.86	671.58	21.85	3.33	6.84
3172_2_SJE_01	12	13.6763319	58.2709974	C	4	9	20.65	5.86	671.58	21.85	3.33	6.84
3172_1_SJE_20	12	13.6763319	58.2709974	C	4	9	20.65	5.86	671.58	21.85	3.33	6.84
3172_2_SJE_35	12	13.6763319	58.2709974	C	4	9	20.65	5.86	671.58	21.85	3.33	6.84
3172_1_SJE_30	12	13.6763319	58.2709974	C	4	9	20.65	5.86	671.58	21.85	3.33	6.84
3172_1_SJE_35	12	13.6763319	58.2709974	C	4	9	20.65	5.86	671.58	21.85	3.33	6.84
3172_2_SJE_32	12	13.6763319	58.2709974	C	4	9	20.65	5.86	671.58	21.85	3.33	6.84
3172_1_SJE_21	12	13.6763319	58.2709974	C	4	9	20.65	5.86	671.58	21.85	3.33	6.84
3172_1_SJE_34	12	13.6763319	58.2709974	C	4	9	20.65	5.86	671.58	21.85	3.33	6.84
3270_2_SJE_09	13	13.8198493	58.5688194	S	4	9	21.36	6.73	590.07	25.64	3.17	6.16
3270_2_SJE_26	13	13.8198493	58.5688194	S	4	9	21.36	6.73	590.07	25.64	3.17	6.16

3270_2_SJE_23	13	13.8198493	58.5688194	S	4	9	21.36	6.73	590.07	25.64	3.17	6.16
3270_2_SJE_06	13	13.8198493	58.5688194	S	4	9	21.36	6.73	590.07	25.64	3.17	6.16
3270_1_SJE_2ter	13	13.8198493	58.5688194	S	4	9	21.36	6.73	590.07	25.64	3.17	6.16
3270_1_SJE_17	13	13.8198493	58.5688194	S	4	9	21.36	6.73	590.07	25.64	3.17	6.16
3270_2_SJE_38	13	13.8198493	58.5688194	S	4	9	21.36	6.73	590.07	25.64	3.17	6.16
3270_1_SJE_20	13	13.8198493	58.5688194	S	4	9	21.36	6.73	590.07	25.64	3.17	6.16
3270_1_SJE_10	13	13.8198493	58.5688194	S	4	9	21.36	6.73	590.07	25.64	3.17	6.16
3270_1_SJE_4ter	13	13.8198493	58.5688194	S	4	9	21.36	6.73	590.07	25.64	3.17	6.16
3270_1_SJE_33	13	13.8198493	58.5688194	S	4	9	21.36	6.73	590.07	25.64	3.17	6.16
3270_1_SJE_24	13	13.8198493	58.5688194	S	4	9	21.36	6.73	590.07	25.64	3.17	6.16
3270_2_SJE_05	13	13.8198493	58.5688194	S	4	9	21.36	6.73	590.07	25.64	3.17	6.16
3270_1_SJE_02	13	13.8198493	58.5688194	S	4	9	21.36	6.73	590.07	25.64	3.17	6.16
3270_1_SJE_03	13	13.8198493	58.5688194	S	4	9	21.36	6.73	590.07	25.64	3.17	6.16
3270_2_SJE_12	13	13.8198493	58.5688194	S	4	9	21.36	6.73	590.07	25.64	3.17	6.16
3270_2_SJE_24	13	13.8198493	58.5688194	S	4	9	21.36	6.73	590.07	25.64	3.17	6.16
4324_2_CH_41	14	14.8775767	59.0665014	C	6	11	21.68	6.17	697.70	25.04	2.67	9.78
4324_2_CH_15	14	14.8775767	59.0665014	C	6	11	21.68	6.17	697.70	25.04	2.67	9.78
4324_2_CH_45	14	14.8775767	59.0665014	C	6	11	21.68	6.17	697.70	25.04	2.67	9.78
4324_1_CH_02	14	14.8775767	59.0665014	C	6	11	21.68	6.17	697.70	25.04	2.67	9.78
4324_1_CH_20	14	14.8775767	59.0665014	C	6	11	21.68	6.17	697.70	25.04	2.67	9.78
4324_2_CH_42	14	14.8775767	59.0665014	C	6	11	21.68	6.17	697.70	25.04	2.67	9.78
4324_2_CH_07	14	14.8775767	59.0665014	C	6	11	21.68	6.17	697.70	25.04	2.67	9.78
4324_2_CH_06	14	14.8775767	59.0665014	C	6	11	21.68	6.17	697.70	25.04	2.67	9.78
4324_1_CH_37	14	14.8775767	59.0665014	C	6	11	21.68	6.17	697.70	25.04	2.67	9.78
4324_1_CH_18	14	14.8775767	59.0665014	C	6	11	21.68	6.17	697.70	25.04	2.67	9.78
4324_1_CH_31	14	14.8775767	59.0665014	C	6	11	21.68	6.17	697.70	25.04	2.67	9.78
4324_2_CH_44	14	14.8775767	59.0665014	C	6	11	21.68	6.17	697.70	25.04	2.67	9.78
4324_1_CH_08	14	14.8775767	59.0665014	C	6	11	21.68	6.17	697.70	25.04	2.67	9.78
4324_1_CH_10	14	14.8775767	59.0665014	C	6	11	21.68	6.17	697.70	25.04	2.67	9.78
4324_2_CH_31	14	14.8775767	59.0665014	C	6	11	21.68	6.17	697.70	25.04	2.67	9.78
4324_1_CH_03	14	14.8775767	59.0665014	C	6	11	21.68	6.17	697.70	25.04	2.67	9.78
4324_1_CH_32	14	14.8775767	59.0665014	C	6	11	21.68	6.17	697.70	25.04	2.67	9.78
4425_2_CH_13	15	15.1920875	59.174183	S	6	11	22.18	6.20	651.23	26.86	2.57	4.60
4425_2_CH_21	15	15.1920875	59.174183	S	6	11	22.18	6.20	651.23	26.86	2.57	4.60
4425_2_CH_02	15	15.1920875	59.174183	S	6	11	22.18	6.20	651.23	26.86	2.57	4.60
4425_2_CH_37	15	15.1920875	59.174183	S	6	11	22.18	6.20	651.23	26.86	2.57	4.60
4425_1_CH_08	15	15.1920875	59.174183	S	6	11	22.18	6.20	651.23	26.86	2.57	4.60
4425_2_CH_20	15	15.1920875	59.174183	S	6	11	22.18	6.20	651.23	26.86	2.57	4.60
4425_2_CH_01	15	15.1920875	59.174183	S	6	11	22.18	6.20	651.23	26.86	2.57	4.60
4425_2_CH_11	15	15.1920875	59.174183	S	6	11	22.18	6.20	651.23	26.86	2.57	4.60
4425_2_CH_05	15	15.1920875	59.174183	S	6	11	22.18	6.20	651.23	26.86	2.57	4.60
4425_1_CH_18	15	15.1920875	59.174183	S	6	11	22.18	6.20	651.23	26.86	2.57	4.60
4425_1_CH_02	15	15.1920875	59.174183	S	6	11	22.18	6.20	651.23	26.86	2.57	4.60
4425_1_CH_36	15	15.1920875	59.174183	S	6	11	22.18	6.20	651.23	26.86	2.57	4.60
4425_2_CH_14	15	15.1920875	59.174183	S	6	11	22.18	6.20	651.23	26.86	2.57	4.60
4723_2_CH_36	16	15.7754657	59.4414299	S	6	12	22.09	5.69	644.58	30.03	2.44	10.97
4723_2_CH_13	16	15.7754657	59.4414299	S	6	12	22.09	5.69	644.58	30.03	2.44	10.97
4723_2_CH_38	16	15.7754657	59.4414299	S	6	12	22.09	5.69	644.58	30.03	2.44	10.97
4723_2_CH_27	16	15.7754657	59.4414299	S	6	12	22.09	5.69	644.58	30.03	2.44	10.97
4723_1_CH_04	16	15.7754657	59.4414299	S	6	12	22.09	5.69	644.58	30.03	2.44	10.97
4723_1_CH_27	16	15.7754657	59.4414299	S	6	12	22.09	5.69	644.58	30.03	2.44	10.97
4723_1_CH_30	16	15.7754657	59.4414299	S	6	12	22.09	5.69	644.58	30.03	2.44	10.97
4723_1_CH_37	16	15.7754657	59.4414299	S	6	12	22.09	5.69	644.58	30.03	2.44	10.97
4723_2_CH_25	16	15.7754657	59.4414299	S	6	12	22.09	5.69	644.58	30.03	2.44	10.97

4723_1_CH_06	16	15.7754657	59.4414299	S	6	12	22.09	5.69	644.58	30.03	2.44	10.97
4723_2_CH_34	16	15.7754657	59.4414299	S	6	12	22.09	5.69	644.58	30.03	2.44	10.97
4723_1_CH_24	16	15.7754657	59.4414299	S	6	12	22.09	5.69	644.58	30.03	2.44	10.97
4723_1_CH_48	16	15.7754657	59.4414299	S	6	12	22.09	5.69	644.58	30.03	2.44	10.97
4723_1_CH_43	16	15.7754657	59.4414299	S	6	12	22.09	5.69	644.58	30.03	2.44	10.97
4723_2_CH_37	16	15.7754657	59.4414299	S	6	12	22.09	5.69	644.58	30.03	2.44	10.97
4723_1_CH_17	16	15.7754657	59.4414299	S	6	12	22.09	5.69	644.58	30.03	2.44	10.97
4723_2_CH_14	16	15.7754657	59.4414299	S	6	12	22.09	5.69	644.58	30.03	2.44	10.97
5534_2_CH_21	17	16.6384769	59.7837369	S	6	13	22.12	5.82	607.94	29.70	2.69	6.96
5534_1_CH_26	17	16.6384769	59.7837369	S	6	13	22.12	5.82	607.94	29.70	2.69	6.96
5534_2_CH_37	17	16.6384769	59.7837369	S	6	13	22.12	5.82	607.94	29.70	2.69	6.96
5534_2_CH_32	17	16.6384769	59.7837369	S	6	13	22.12	5.82	607.94	29.70	2.69	6.96
5534_1_CH_08	17	16.6384769	59.7837369	S	6	13	22.12	5.82	607.94	29.70	2.69	6.96
5534_1_CH_28	17	16.6384769	59.7837369	S	6	13	22.12	5.82	607.94	29.70	2.69	6.96
5534_2_CH_12	17	16.6384769	59.7837369	S	6	13	22.12	5.82	607.94	29.70	2.69	6.96
5534_2_CH_36	17	16.6384769	59.7837369	S	6	13	22.12	5.82	607.94	29.70	2.69	6.96
5534_1_CH_24	17	16.6384769	59.7837369	S	6	13	22.12	5.82	607.94	29.70	2.69	6.96
5534_1_CH_36	17	16.6384769	59.7837369	S	6	13	22.12	5.82	607.94	29.70	2.69	6.96
5811_1_CH_19	18	16.8461685	59.6732015	C	6	12	22.20	6.04	568.07	29.92	2.71	7.47
5811_2_CH_20	18	16.8461685	59.6732015	C	6	12	22.20	6.04	568.07	29.92	2.71	7.47
5811_2_CH_33	18	16.8461685	59.6732015	C	6	12	22.20	6.04	568.07	29.92	2.71	7.47
5811_1_CH_21	18	16.8461685	59.6732015	C	6	12	22.20	6.04	568.07	29.92	2.71	7.47
5811_1_CH_34	18	16.8461685	59.6732015	C	6	12	22.20	6.04	568.07	29.92	2.71	7.47
5811_1_CH_29	18	16.8461685	59.6732015	C	6	12	22.20	6.04	568.07	29.92	2.71	7.47
5811_1_CH_01	18	16.8461685	59.6732015	C	6	12	22.20	6.04	568.07	29.92	2.71	7.47
5811_2_CH_40	18	16.8461685	59.6732015	C	6	12	22.20	6.04	568.07	29.92	2.71	7.47
5811_1_CH_07	18	16.8461685	59.6732015	C	6	12	22.20	6.04	568.07	29.92	2.71	7.47
5811_1_CH_28	18	16.8461685	59.6732015	C	6	12	22.20	6.04	568.07	29.92	2.71	7.47
5811_2_CH_31	18	16.8461685	59.6732015	C	6	12	22.20	6.04	568.07	29.92	2.71	7.47
5811_1_CH_41	18	16.8461685	59.6732015	C	6	12	22.20	6.04	568.07	29.92	2.71	7.47
5811_1_CH_12	18	16.8461685	59.6732015	C	6	12	22.20	6.04	568.07	29.92	2.71	7.47
5811_2_CH_32	18	16.8461685	59.6732015	C	6	12	22.20	6.04	568.07	29.92	2.71	7.47
5811_2_CH_12	18	16.8461685	59.6732015	C	6	12	22.20	6.04	568.07	29.92	2.71	7.47
6553_2_CH_13	19	17.3802538	59.6915473	C	6	13	21.75	5.96	555.37	28.71	2.72	4.73
6553_2_CH_19	19	17.3802538	59.6915473	C	6	13	21.75	5.96	555.37	28.71	2.72	4.73
6553_2_CH_01	19	17.3802538	59.6915473	C	6	13	21.75	5.96	555.37	28.71	2.72	4.73
6553_1_CH_37	19	17.3802538	59.6915473	C	6	13	21.75	5.96	555.37	28.71	2.72	4.73
6553_2_CH_08	19	17.3802538	59.6915473	C	6	13	21.75	5.96	555.37	28.71	2.72	4.73
6553_2_CH_27	19	17.3802538	59.6915473	C	6	13	21.75	5.96	555.37	28.71	2.72	4.73
6553_1_CH_05	19	17.3802538	59.6915473	C	6	13	21.75	5.96	555.37	28.71	2.72	4.73
6553_2_CH_03	19	17.3802538	59.6915473	C	6	13	21.75	5.96	555.37	28.71	2.72	4.73
6553_2_CH_18	19	17.3802538	59.6915473	C	6	13	21.75	5.96	555.37	28.71	2.72	4.73
6553_1_CH_02	19	17.3802538	59.6915473	C	6	13	21.75	5.96	555.37	28.71	2.72	4.73
6553_1_CH_41	19	17.3802538	59.6915473	C	6	13	21.75	5.96	555.37	28.71	2.72	4.73
6553_1_CH_36	19	17.3802538	59.6915473	C	6	13	21.75	5.96	555.37	28.71	2.72	4.73
6553_1_CH_34	19	17.3802538	59.6915473	C	6	13	21.75	5.96	555.37	28.71	2.72	4.73
6553_1_CH_04	19	17.3802538	59.6915473	C	6	13	21.75	5.96	555.37	28.71	2.72	4.73
6553_2_CH_02	19	17.3802538	59.6915473	C	6	13	21.75	5.96	555.37	28.71	2.72	4.73
6553_1_CH_45	19	17.3802538	59.6915473	C	6	13	21.75	5.96	555.37	28.71	2.72	4.73
6553_1_CH_38	19	17.3802538	59.6915473	C	6	13	21.75	5.96	555.37	28.71	2.72	4.73

Percentage SNH	Uncultivated agricultural field borders km	Percentage agricultural land cover	Percentage urban cover	Percent age forest cover	ITD	Body weight	Body length	Small wing length	Big wing length	Small wing area	Big wing area	Wing pair area total	Wing loading
0.00	244.77	0.77	0.09	0.03	3.67	0.13	12.41	6.25	9.11	7.07	16.90	2.40	0.05
0.00	244.77	0.77	0.09	0.03	3.00	0.13	13.17	6.59	10.08	7.76	18.42	2.62	0.05
0.00	244.77	0.77	0.09	0.03	4.37	0.30	14.49	7.79	10.87	12.06	24.52	3.66	0.08
0.00	244.77	0.77	0.09	0.03	3.45	0.14	11.61	6.85	10.19	8.67	20.62	2.93	0.05
0.00	244.77	0.77	0.09	0.03	4.90	0.29	15.80	8.14	12.44	14.22	30.16	4.44	0.07
0.00	244.77	0.77	0.09	0.03	3.90	0.18	14.77	6.95	10.68	9.99	21.93	3.19	0.06
0.00	244.77	0.77	0.09	0.03	3.26	0.15	13.05	7.04	10.23	8.75	19.82	2.86	0.05
0.00	244.77	0.77	0.09	0.03	3.69	0.19	13.12	6.80	10.03	8.82	20.04	2.89	0.06
0.00	244.77	0.77	0.09	0.03	4.50	0.23	14.29	7.82	11.82	12.71	25.95	3.87	0.06
0.00	244.77	0.77	0.09	0.03	3.70	0.18	13.75	7.36	10.72	10.29	20.72	3.10	0.06
0.00	244.77	0.77	0.09	0.03	4.86	0.23	15.22	8.04	11.77	13.70	27.52	4.11	0.06
0.00	244.77	0.77	0.09	0.03	4.84	0.22	14.35	7.87	11.22	12.26	26.21	3.85	0.06
0.00	244.77	0.77	0.09	0.03	3.90	0.19	13.34	6.78	10.45	8.65	22.12	3.08	0.06
0.00	244.77	0.77	0.09	0.03	4.70	0.21	16.43	7.76	10.84	11.72	24.30	3.60	0.06
0.00	244.77	0.77	0.09	0.03	4.70	0.27	14.12	8.69	11.27	11.74	27.34	3.91	0.07
0.00	244.77	0.77	0.09	0.03	3.84	0.17	15.01	7.58	11.21	10.84	24.35	3.52	0.05
0.00	229.94	0.85	0.04	0.04	4.86	0.15	14.61	7.06	10.70	7.22	23.40	3.39	0.05
0.00	229.94	0.85	0.04	0.04	4.00	0.17	14.67	8.34	9.68	8.41	19.59	2.93	0.06
0.00	229.94	0.85	0.04	0.04	5.26	0.15	16.01	8.32	12.01	14.24	30.43	4.47	0.03
0.00	229.94	0.85	0.04	0.04	4.07	0.16	15.40	6.73	10.11	9.35	21.05	3.04	0.05
0.00	229.94	0.85	0.04	0.04	4.20	0.29	15.64	8.10	11.28	11.73	25.14	3.70	0.08
0.00	229.94	0.85	0.04	0.04	4.36	0.21	12.94	7.11	10.72	9.88	22.86	3.27	0.06
0.00	229.94	0.85	0.04	0.04	4.65	0.20	13.96	7.54	11.39	10.82	25.14	3.60	0.06
0.00	229.94	0.85	0.04	0.04	4.71	0.25	13.84	7.30	10.50	9.62	22.75	3.24	0.08
0.00	229.94	0.85	0.04	0.04	3.35	0.17	12.82	6.61	9.35	8.28	18.49	2.68	0.06
0.00	229.94	0.85	0.04	0.04	3.71	0.15	13.85	6.49	10.70	8.21	23.40	3.39	0.04
0.00	229.94	0.85	0.04	0.04	4.53	0.22	14.73	7.60	11.48	11.34	26.90	3.82	0.06
0.00	229.94	0.85	0.04	0.04	4.48	0.19	14.39	7.38	11.18	11.86	27.08	3.89	0.05
0.00	229.94	0.85	0.04	0.04	4.43	0.19	14.81	7.71	11.36	11.23	25.76	3.70	0.05
0.00	229.94	0.85	0.04	0.04	3.85	0.17	12.65	7.60	10.42	10.65	22.99	3.36	0.05
0.00	229.94	0.85	0.04	0.04	3.55	0.16	12.89	6.87	10.10	8.65	19.78	2.84	0.06
0.00	229.94	0.85	0.04	0.04	4.06	0.18	13.96	7.51	10.39	10.29	21.59	3.19	0.06
0.01	262.34	0.89	0.04	0.02	3.88	0.14	11.70	6.80	10.60	8.95	22.28	3.23	0.05
0.01	262.34	0.89	0.04	0.02	3.37	0.14	12.35	6.62	9.70	8.85	19.43	2.83	0.05
0.01	262.34	0.89	0.04	0.02	3.96	0.17	13.03	7.37	10.14	9.99	21.56	3.16	0.05
0.01	262.34	0.89	0.04	0.02	3.67	0.22	15.08	7.21	10.60	9.45	21.93	3.14	0.07
0.01	262.34	0.89	0.04	0.02	3.45	0.11	13.06	6.63	9.10	7.61	18.24	2.58	0.04
0.01	262.34	0.89	0.04	0.02	3.76	0.13	13.11	6.41	9.50	7.95	18.67	2.66	0.05
0.01	262.34	0.89	0.04	0.02	4.28	0.21	14.40	8.78	13.09	14.41	31.84	4.63	0.05
0.01	262.34	0.89	0.04	0.02	3.84	0.21	13.72	7.78	11.00	11.18	22.15	3.33	0.06
0.01	262.34	0.89	0.04	0.02	4.10	0.22	15.06	8.26	11.82	11.98	28.23	4.02	0.05
0.01	262.34	0.89	0.04	0.02	4.18	0.21	14.45	8.00	11.97	11.59	26.63	3.82	0.05
0.01	262.34	0.89	0.04	0.02	3.18	0.16	10.76	5.63	8.52	6.59	14.39	2.10	0.07
0.01	262.34	0.89	0.04	0.02	3.99	0.20	13.54	7.14	10.07	9.52	19.60	2.91	0.07
0.01	262.34	0.89	0.04	0.02	3.41	0.13	13.01	6.33	9.67	7.93	17.40	2.53	0.05
0.01	262.34	0.89	0.04	0.02	3.80	0.16	16.04	8.76	12.77	13.31	29.42	4.27	0.04
0.01	262.34	0.89	0.04	0.02	4.01	0.18	14.43	7.71	10.99	10.99	24.55	3.55	0.05

0.01	262.34	0.89	0.04	0.02	4.05	0.15	12.56	7.04	10.09	8.42	20.11	2.85	0.05
0.01	262.34	0.89	0.04	0.02	3.71	0.14	13.33	6.28	10.60	7.34	22.28	3.23	0.05
0.05	294.62	0.80	0.04	0.04	3.76	0.22	13.70	7.35	10.34	10.52	22.73	3.33	0.07
0.05	294.62	0.80	0.04	0.04	3.19	0.11	13.09	6.10	9.45	8.03	18.03	2.61	0.04
0.05	294.62	0.80	0.04	0.04	3.60	0.12	12.41	6.29	8.93	6.95	16.59	2.35	0.05
0.05	294.62	0.80	0.04	0.04	3.68	0.12	11.69	6.57	9.46	7.36	16.63	2.40	0.05
0.05	294.62	0.80	0.04	0.04	3.60	0.19	12.88	7.40	10.56	10.26	21.86	3.21	0.06
0.05	294.62	0.80	0.04	0.04	3.43	0.15	11.35	5.94	8.71	6.21	15.31	2.15	0.07
0.05	294.62	0.80	0.04	0.04	4.70	0.19	13.87	7.59	11.30	10.78	22.50	3.33	0.06
0.05	294.62	0.80	0.04	0.04	3.63	0.13	13.62	6.58	9.20	6.87	18.12	2.50	0.05
0.05	294.62	0.80	0.04	0.04	4.25	0.21	13.99	7.94	11.04	11.28	25.15	3.64	0.06
0.05	294.62	0.80	0.04	0.04	4.59	0.23	15.45	8.24	12.20	12.73	28.33	4.11	0.06
0.05	294.62	0.80	0.04	0.04	3.42	0.13	11.33	5.85	8.45	6.08	14.24	2.03	0.06
0.05	294.62	0.80	0.04	0.04	3.97	0.19	13.99	6.94	10.57	10.23	22.26	3.25	0.06
0.05	294.62	0.80	0.04	0.04	4.08	0.22	13.67	7.18	11.14	11.55	23.57	3.51	0.06
0.05	294.62	0.80	0.04	0.04	4.00	0.26	16.26	9.14	13.74	16.13	35.64	5.18	0.05
0.05	294.62	0.80	0.04	0.04	3.20	0.11	12.02	6.24	8.03	6.98	14.75	2.17	0.05
0.05	294.62	0.80	0.04	0.04	4.31	0.19	15.40	7.90	11.15	12.29	28.71	4.10	0.05
0.05	294.62	0.80	0.04	0.04	3.65	0.12	14.19	7.08	10.27	9.64	21.53	3.12	0.04
0.06	302.24	0.45	0.03	0.40	4.28	0.21	14.13	7.72	10.21	11.21	22.97	3.42	0.06
0.06	302.24	0.45	0.03	0.40	4.45	0.19	14.30	7.51	10.94	9.99	23.54	3.35	0.06
0.06	302.24	0.45	0.03	0.40	4.63	0.29	16.12	7.80	11.27	13.05	27.80	4.08	0.07
0.06	302.24	0.45	0.03	0.40	4.49	0.31	15.08	8.29	11.69	11.97	28.06	4.00	0.08
0.06	302.24	0.45	0.03	0.40	3.90	0.19	14.23	7.42	10.10	9.83	20.72	3.06	0.06
0.06	302.24	0.45	0.03	0.40	3.17	0.13	12.50	6.15	9.44	7.38	17.51	2.49	0.05
0.06	302.24	0.45	0.03	0.40	4.22	0.19	13.20	7.25	10.84	9.29	22.33	3.16	0.06
0.06	302.24	0.45	0.03	0.40	3.84	0.21	14.18	7.26	10.80	9.93	22.26	3.22	0.06
0.06	302.24	0.45	0.03	0.40	4.55	0.20	14.65	7.88	11.81	11.61	25.64	3.73	0.05
0.06	302.24	0.45	0.03	0.40	4.26	0.20	14.96	7.88	11.11	10.94	25.10	3.60	0.06
0.06	302.24	0.45	0.03	0.40	4.71	0.29	15.01	8.19	12.30	13.00	29.57	4.26	0.07
0.06	302.24	0.45	0.03	0.40	4.27	0.18	14.89	7.62	11.28	11.24	24.75	3.60	0.05
0.06	302.24	0.45	0.03	0.40	4.92	0.27	15.13	8.05	11.79	11.31	26.88	3.82	0.07
0.06	302.24	0.45	0.03	0.40	3.18	0.14	14.91	7.55	9.88	10.61	19.26	3.47	0.04
0.06	302.24	0.45	0.03	0.40	4.41	0.20	14.70	7.12	10.67	9.30	21.89	3.12	0.06
0.06	302.24	0.45	0.03	0.40	3.41	0.20	14.06	6.76	10.14	8.61	20.63	2.92	0.07
0.06	302.24	0.45	0.03	0.40	4.59	0.26	16.29	7.96	11.46	11.53	26.04	3.76	0.07
0.06	399.90	0.80	0.05	0.05	4.08	0.21	15.12	7.62	11.40	11.51	25.97	3.75	0.06
0.06	399.90	0.80	0.05	0.05	4.78	0.31	15.77	7.28	11.51	12.66	28.32	4.10	0.08
0.06	399.90	0.80	0.05	0.05	4.37	0.20	15.28	7.30	10.82	10.93	24.44	3.54	0.06
0.06	399.90	0.80	0.05	0.05	3.69	0.17	13.60	6.76	9.28	8.82	18.95	2.78	0.06
0.06	399.90	0.80	0.05	0.05	4.00	0.18	13.64	7.35	10.99	9.47	22.51	3.20	0.06
0.06	399.90	0.80	0.05	0.05	3.69	0.20	14.76	7.59	11.47	11.34	24.74	3.61	0.06
0.06	399.90	0.80	0.05	0.05	3.59	0.18	12.30	7.20	10.18	9.05	20.30	2.94	0.06
0.06	399.90	0.80	0.05	0.05	4.41	0.19	14.35	7.69	10.42	10.42	22.84	3.33	0.06
0.06	399.90	0.80	0.05	0.05	4.06	0.15	12.79	6.68	9.40	7.66	18.16	2.58	0.06
0.06	399.90	0.80	0.05	0.05	4.53	0.19	13.29	7.28	10.47	9.30	20.28	2.96	0.06
0.06	399.90	0.80	0.05	0.05	4.45	0.19	14.69	6.76	10.81	10.15	22.32	3.25	0.06
0.06	399.90	0.80	0.05	0.05	4.43	0.24	15.43	7.77	10.52	10.67	24.19	3.49	0.07
0.06	399.90	0.80	0.05	0.05	4.16	0.19	13.30	7.16	10.65	9.73	22.78	3.25	0.06
0.06	399.90	0.80	0.05	0.05	4.61	0.21	15.37	7.46	10.83	9.41	24.68	3.41	0.06
0.06	399.90	0.80	0.05	0.05	3.96	0.13	13.10	7.20	8.94	8.08	17.20	2.53	0.05
0.06	399.90	0.80	0.05	0.05	4.24	0.19	14.27	7.21	10.88	9.69	23.43	3.31	0.06
0.06	399.90	0.80	0.05	0.05	4.31	0.22	15.05	7.16	10.77	9.86	22.45	3.23	0.07
0.05	344.02	0.80	0.05	0.03	4.43	0.21	14.52	7.54	11.17	11.08	25.48	3.69	0.06

0.05	344.02	0.80	0.05	0.03	5.28	0.24	15.38	7.94	11.01	12.20	27.30	3.95	0.06
0.05	344.02	0.80	0.05	0.03	3.83	0.22	14.89	7.59	10.63	10.48	23.80	3.43	0.06
0.05	344.02	0.80	0.05	0.03	4.81	0.21	14.94	8.20	11.38	12.58	26.32	3.89	0.06
0.05	344.02	0.80	0.05	0.03	4.92	0.24	14.40	8.23	11.92	12.33	27.32	3.97	0.06
0.05	344.02	0.80	0.05	0.03	5.41	0.23	15.47	8.42	11.58	12.76	27.71	4.05	0.06
0.05	344.02	0.80	0.05	0.03	4.30	0.20	14.76	7.58	11.17	10.34	24.01	3.44	0.06
0.05	344.02	0.80	0.05	0.03	4.75	0.22	13.73	7.89	11.63	12.66	27.21	3.99	0.06
0.05	344.02	0.80	0.05	0.03	4.49	0.20	15.03	7.55	10.90	11.36	24.59	3.60	0.06
0.05	344.02	0.80	0.05	0.03	4.71	0.20	15.22	7.96	11.59	12.06	27.46	3.95	0.05
0.05	344.02	0.80	0.05	0.03	4.65	0.25	13.73	7.74	11.80	12.02	27.24	3.93	0.06
0.05	344.02	0.80	0.05	0.03	4.09	0.18	15.18	7.69	11.36	10.47	23.67	3.41	0.05
0.05	344.02	0.80	0.05	0.03	5.14	0.20	15.07	7.70	11.12	11.18	25.92	3.71	0.05
0.05	344.02	0.80	0.05	0.03	4.75	0.21	14.81	7.17	10.79	10.30	23.60	3.39	0.06
0.05	344.02	0.80	0.05	0.03	4.80	0.22	15.54	8.00	10.81	11.23	25.83	3.71	0.06
0.05	344.02	0.80	0.05	0.03	4.77	0.20	14.42	8.16	11.16	12.48	26.10	3.86	0.05
0.05	344.02	0.80	0.05	0.03	4.26	0.15	13.31	6.74	9.91	8.94	19.64	2.86	0.05
0.06	318.01	0.44	0.04	0.35	4.40	0.16	14.02	6.87	10.04	9.05	21.56	3.06	0.05
0.06	318.01	0.44	0.04	0.35	4.30	0.18	14.65	7.28	10.96	10.65	22.14	3.28	0.05
0.06	318.01	0.44	0.04	0.35	4.50	0.26	16.26	8.24	11.87	13.01	30.51	4.35	0.06
0.06	318.01	0.44	0.04	0.35	4.36	0.20	14.73	7.03	10.61	9.82	21.07	3.09	0.07
0.06	318.01	0.44	0.04	0.35	3.86	0.15	11.71	6.47	10.16	6.67	19.13	2.58	0.06
0.06	318.01	0.44	0.04	0.35	5.37	0.26	17.11	8.79	13.16	15.58	33.39	4.90	0.05
0.06	318.01	0.44	0.04	0.35	4.73	0.21	13.69	7.45	11.21	9.28	23.26	3.25	0.07
0.06	318.01	0.44	0.04	0.35	3.80	0.11	11.82	6.58	11.39	6.76	24.29	3.69	0.03
0.06	318.01	0.44	0.04	0.35	4.43	0.36	16.46	8.60	12.82	13.45	29.99	4.34	0.08
0.06	318.01	0.44	0.04	0.35	4.61	0.24	15.27	8.06	11.73	12.77	25.74	3.85	0.06
0.06	318.01	0.44	0.04	0.35	4.08	0.29	15.61	8.84	13.13	15.57	32.12	4.77	0.06
0.06	318.01	0.44	0.04	0.35	4.99	0.23	13.76	8.02	11.24	11.19	24.38	3.56	0.06
0.06	318.01	0.44	0.04	0.35	4.26	0.16	12.15	7.30	10.58	9.31	21.30	3.06	0.05
0.06	318.01	0.44	0.04	0.35	4.10	0.31	15.93	8.25	12.17	13.13	29.72	4.29	0.07
0.06	318.01	0.44	0.04	0.35	4.31	0.19	14.54	7.33	10.20	9.80	21.30	3.11	0.06
0.06	318.01	0.44	0.04	0.35	4.73	0.20	13.95	7.96	11.79	26.40	11.52	3.79	0.05
0.06	318.01	0.44	0.04	0.35	3.75	0.16	13.99	7.68	10.65	12.03	21.52	3.69	0.04
0.01	191.41	0.41	0.03	0.47	4.26	0.19	14.24	7.35	10.53	10.92	24.12	3.24	0.06
0.01	191.41	0.41	0.03	0.47	4.02	0.14	12.45	6.84	9.59	8.66	19.77	2.84	0.05
0.01	191.41	0.41	0.03	0.47	4.31	0.21	15.09	7.46	10.83	10.72	20.27	3.10	0.07
0.01	191.41	0.41	0.03	0.47	4.84	0.24	15.80	7.38	10.86	10.84	25.75	3.66	0.07
0.01	191.41	0.41	0.03	0.47	4.27	0.19	13.57	7.27	10.53	9.23	24.12	3.24	0.06
0.01	191.41	0.41	0.03	0.47	4.84	0.25	14.40	7.79	11.44	11.18	25.49	3.67	0.07
0.01	191.41	0.41	0.03	0.47	5.00	0.17	13.86	7.17	10.16	10.29	21.53	3.18	0.05
0.01	191.41	0.41	0.03	0.47	4.71	0.23	15.34	7.58	10.72	10.61	23.00	3.36	0.07
0.01	191.41	0.41	0.03	0.47	3.39	0.13	11.66	6.29	8.71	7.17	16.58	2.38	0.05
0.01	191.41	0.41	0.03	0.47	5.18	0.25	15.01	8.75	12.28	13.38	30.44	4.38	0.06
0.01	191.41	0.41	0.03	0.47	4.30	0.13	12.38	6.40	9.84	7.54	18.15	2.57	0.05
0.01	191.41	0.41	0.03	0.47	4.40	0.19	14.57	6.76	10.19	8.51	19.94	2.85	0.07
0.01	191.41	0.41	0.03	0.47	3.95	0.21	14.30	7.99	10.52	10.35	23.72	3.41	0.06
0.01	191.41	0.41	0.03	0.47	4.47	0.24	14.30	8.20	12.05	12.12	26.54	3.87	0.06
0.01	191.41	0.41	0.03	0.47	4.57	0.18	14.83	7.78	10.98	11.65	24.33	3.60	0.05
0.01	191.41	0.41	0.03	0.47	3.39	0.13	12.32	5.89	8.80	6.44	16.72	2.32	0.05
0.00	237.42	0.50	0.03	0.42	3.82	0.16	15.18	7.19	10.04	9.85	21.60	3.15	0.05
0.00	237.42	0.50	0.03	0.42	5.25	0.24	14.16	7.41	10.64	10.31	22.82	3.31	0.07
0.00	237.42	0.50	0.03	0.42	4.16	0.20	12.73	6.70	9.71	8.15	17.41	2.56	0.08
0.00	237.42	0.50	0.03	0.42	4.97	0.16	15.96	7.60	11.85	8.96	23.30	3.23	0.05
0.00	237.42	0.50	0.03	0.42	4.51	0.27	15.99	7.53	11.30	11.37	23.17	3.45	0.08

0.00	237.42	0.50	0.03	0.42	4.90	0.16	13.66	7.50	10.19	10.03	25.07	3.51	0.05
0.00	237.42	0.50	0.03	0.42	5.00	0.22	14.54	7.38	11.42	11.23	25.51	3.68	0.06
0.00	237.42	0.50	0.03	0.42	4.14	0.18	14.41	7.17	10.69	9.79	21.21	3.10	0.06
0.00	237.42	0.50	0.03	0.42	4.14	0.33	16.33	8.80	13.03	15.47	31.92	4.74	0.07
0.00	237.42	0.50	0.03	0.42	4.20	0.19	14.37	7.61	11.12	10.99	25.16	3.62	0.05
0.00	237.42	0.50	0.03	0.42	4.86	0.25	14.62	8.34	12.57	14.00	29.52	4.35	0.06
0.00	237.42	0.50	0.03	0.42	3.69	0.15	13.10	6.59	10.12	7.51	17.41	2.49	0.06
0.00	237.42	0.50	0.03	0.42	4.82	0.16	14.78	6.67	10.12	8.70	19.84	2.85	0.05
0.00	237.42	0.50	0.03	0.42	4.41	0.21	14.06	7.39	10.87	10.27	22.98	3.33	0.06
0.00	237.42	0.50	0.03	0.42	3.92	0.11	12.47	6.25	9.42	6.76	16.72	2.35	0.05
0.00	237.42	0.50	0.03	0.42	4.67	0.25	15.23	7.90	10.38	10.49	23.12	3.36	0.07
0.00	237.42	0.50	0.03	0.42	4.22	0.18	14.74	7.54	11.25	10.76	23.84	3.46	0.05
0.07	306.53	0.49	0.03	0.31	4.75	0.26	16.44	8.01	12.17	11.98	27.95	3.99	0.07
0.07	306.53	0.49	0.03	0.31	4.77	0.22	15.65	7.63	10.78	11.01	24.22	3.52	0.06
0.07	306.53	0.49	0.03	0.31	4.37	0.18	13.77	7.37	10.66	9.75	22.17	3.19	0.06
0.07	306.53	0.49	0.03	0.31	4.10	0.19	14.14	7.59	10.37	9.30	19.78	2.91	0.07
0.07	306.53	0.49	0.03	0.31	4.18	0.23	14.32	7.45	10.46	9.60	23.16	3.28	0.07
0.07	306.53	0.49	0.03	0.31	4.68	0.35	14.50	8.36	12.49	13.76	28.64	4.24	0.08
0.07	306.53	0.49	0.03	0.31	3.88	0.21	14.38	7.49	10.86	10.14	21.98	3.21	0.07
0.07	306.53	0.49	0.03	0.31	5.08	0.22	14.98	7.99	11.69	12.46	26.90	3.94	0.06
0.07	306.53	0.49	0.03	0.31	4.47	0.20	14.28	7.33	11.18	10.57	24.95	3.55	0.06
0.07	306.53	0.49	0.03	0.31	4.22	0.24	15.47	7.42	10.70	10.81	23.66	3.45	0.07
0.07	306.53	0.49	0.03	0.31	5.14	0.26	15.16	8.37	12.06	13.44	28.68	4.21	0.06
0.07	306.53	0.49	0.03	0.31	4.63	0.22	14.91	7.50	10.90	10.51	23.47	3.40	0.06
0.07	306.53	0.49	0.03	0.31	4.00	0.17	13.36	6.91	9.79	9.02	19.61	2.86	0.06
0.07	306.53	0.49	0.03	0.31	4.25	0.17	14.11	7.13	10.66	9.80	21.71	3.15	0.05
0.07	332.76	0.44	0.05	0.35	3.41	0.18	13.96	6.82	9.76	8.58	19.66	2.82	0.07
0.07	332.76	0.44	0.05	0.35	4.16	0.20	14.62	7.39	10.32	10.82	23.95	3.48	0.06
0.07	332.76	0.44	0.05	0.35	3.88	0.15	13.56	7.03	9.57	8.62	19.54	2.82	0.06
0.07	332.76	0.44	0.05	0.35	3.70	0.18	15.02	7.25	10.64	9.31	21.07	3.04	0.06
0.07	332.76	0.44	0.05	0.35	4.49	0.24	13.55	7.86	11.89	10.68	26.31	3.70	0.06
0.07	332.76	0.44	0.05	0.35	3.57	0.15	13.71	7.78	11.87	10.68	26.31	3.70	0.04
0.07	332.76	0.44	0.05	0.35	4.28	0.20	14.42	7.28	11.34	11.21	22.85	3.41	0.06
0.07	332.76	0.44	0.05	0.35	4.39	0.21	13.72	7.74	11.17	9.32	24.30	3.36	0.06
0.07	332.76	0.44	0.05	0.35	3.88	0.15	12.63	7.04	9.84	8.92	19.77	2.87	0.05
0.07	332.76	0.44	0.05	0.35	4.00	0.16	13.25	7.12	10.42	10.00	23.05	3.31	0.05
0.07	332.76	0.44	0.05	0.35	4.95	0.22	14.89	7.93	11.54	12.29	26.35	3.86	0.06
0.07	332.76	0.44	0.05	0.35	4.39	0.19	15.37	7.61	10.86	10.34	22.73	3.31	0.06
0.07	332.76	0.44	0.05	0.35	3.98	0.15	12.32	6.68	9.61	8.64	18.77	2.74	0.05
0.07	332.76	0.44	0.05	0.35	4.00	0.19	15.23	7.58	10.76	10.16	22.63	3.28	0.06
0.07	332.76	0.44	0.05	0.35	4.47	0.16	13.23	7.26	10.58	8.40	20.88	2.93	0.05
0.07	332.76	0.44	0.05	0.35	3.84	0.24	13.67	7.55	11.20	11.09	24.13	3.52	0.07
0.07	332.76	0.44	0.05	0.35	4.35	0.24	14.46	8.44	12.96	13.82	30.61	4.44	0.05
0.01	215.85	0.60	0.03	0.30	14.4	0.15	15.46	6.88	10.38	9.53	21.02	3.06	0.05
0.01	215.85	0.60	0.03	0.30	4.49	0.22	15.26	7.08	10.42	10.09	22.31	3.24	0.07
0.01	215.85	0.60	0.03	0.30	4.34	0.20	16.46	7.61	11.47	11.81	26.24	3.81	0.05
0.01	215.85	0.60	0.03	0.30	4.04	0.18	15.12	7.98	10.67	11.86	24.65	3.65	0.05
0.01	215.85	0.60	0.03	0.30	3.73	0.13	12.11	6.58	9.78	8.15	19.45	2.76	0.05
0.01	215.85	0.60	0.03	0.30	3.86	0.14	11.66	6.95	10.02	8.62	18.71	2.73	0.05
0.01	215.85	0.60	0.03	0.30	3.90	0.15	13.42	7.29	10.55	9.91	22.04	3.19	0.05
0.01	215.85	0.60	0.03	0.30	4.22	0.15	12.92	7.64	10.44	10.04	22.55	3.26	0.05
0.01	215.85	0.60	0.03	0.30	4.01	0.16	13.47	7.29	10.55	9.91	22.04	3.19	0.05
0.01	215.85	0.60	0.03	0.30	3.49	0.13	11.88	6.10	9.06	6.58	16.09	2.27	0.06
0.01	215.85	0.60	0.03	0.30	4.20	0.15	12.79	7.19	10.75	9.26	20.74	3.00	0.05

0.01	215.85	0.60	0.03	0.30	3.73	0.17	11.90	6.83	9.72	8.27	19.96	2.82	0.06
0.01	215.85	0.60	0.03	0.30	3.61	0.15	12.43	7.04	9.71	9.28	18.95	2.82	0.05
0.01	215.85	0.60	0.03	0.30	3.94	0.20	14.35	7.94	11.84	11.47	26.36	3.78	0.05
0.01	215.85	0.60	0.03	0.30	4.37	0.20	14.09	7.54	10.70	10.16	21.64	3.18	0.06
0.01	215.85	0.60	0.03	0.30	4.47	0.18	16.40	8.22	12.00	12.32	26.29	3.86	0.05
0.01	215.85	0.60	0.03	0.30	4.52	0.31	15.95	7.79	11.33	11.21	25.60	3.68	0.08
0.07	283.22	0.49	0.03	0.28	5.10	0.24	16.90	8.24	11.73	13.10	28.59	4.17	0.06
0.07	283.22	0.49	0.03	0.28	4.53	0.25	15.75	7.74	11.16	11.85	26.29	3.81	0.07
0.07	283.22	0.49	0.03	0.28	4.51	0.22	15.39	8.18	11.74	12.62	27.03	3.97	0.06
0.07	283.22	0.49	0.03	0.28	4.35	0.23	14.65	8.28	12.22	12.14	27.49	3.96	0.06
0.07	283.22	0.49	0.03	0.28	3.66	0.21	12.64	6.76	10.40	9.47	21.04	3.05	0.07
0.07	283.22	0.49	0.03	0.28	4.73	0.23	15.41	7.84	11.53	12.85	27.57	4.04	0.06
0.07	283.22	0.49	0.03	0.28	4.10	0.17	14.45	7.43	11.00	10.64	22.76	3.34	0.05
0.07	283.22	0.49	0.03	0.28	3.82	0.20	13.65	7.79	11.21	11.21	24.90	3.61	0.06
0.07	283.22	0.49	0.03	0.28	4.51	0.14	14.97	7.76	11.04	11.59	25.60	3.72	0.04
0.07	283.22	0.49	0.03	0.28	4.10	0.20	12.88	7.67	10.53	9.93	22.52	3.25	0.06
0.07	283.22	0.49	0.03	0.28	4.63	0.23	14.86	7.70	11.05	11.59	25.60	3.72	0.06
0.07	283.22	0.49	0.03	0.28	4.02	0.17	13.41	7.43	10.64	10.22	22.41	3.26	0.05
0.07	283.22	0.49	0.03	0.28	3.60	0.21	14.26	7.16	10.14	8.92	20.17	2.91	0.07
0.07	283.22	0.49	0.03	0.28	3.47	0.14	12.26	6.36	9.50	7.18	14.95	2.21	0.06
0.07	283.22	0.49	0.03	0.28	4.71	0.23	15.34	7.42	10.79	11.30	23.83	3.51	0.07
0.07	283.22	0.49	0.03	0.28	3.69	0.14	12.68	6.87	10.42	8.05	19.29	2.73	0.05
0.07	283.22	0.49	0.03	0.28	4.12	0.18	13.39	6.77	9.43	9.15	19.60	2.88	0.06
0.01	201.84	0.54	0.03	0.31	4.54	0.23	16.28	7.84	12.24	13.34	28.16	4.15	0.06
0.01	201.84	0.54	0.03	0.31	3.97	0.22	15.85	7.64	11.42	12.48	26.70	3.92	0.06
0.01	201.84	0.54	0.03	0.31	4.63	0.28	16.17	7.27	10.74	11.63	26.07	3.77	0.07
0.01	201.84	0.54	0.03	0.31	4.28	0.17	15.54	7.54	11.18	11.01	24.03	3.50	0.05
0.01	201.84	0.54	0.03	0.31	3.75	0.16	13.54	6.96	10.81	9.87	22.40	3.23	0.05
0.01	201.84	0.54	0.03	0.31	4.83	0.32	15.46	8.31	12.49	14.48	31.15	4.56	0.07
0.01	201.84	0.54	0.03	0.31	4.16	0.18	13.53	6.17	9.79	7.79	18.70	2.65	0.07
0.01	201.84	0.54	0.03	0.31	4.26	0.24	14.15	7.24	10.54	10.29	22.93	3.32	0.07
0.01	201.84	0.54	0.03	0.31	4.07	0.25	13.56	7.57	11.02	10.52	25.54	3.61	0.07
0.01	201.84	0.54	0.03	0.31	4.71	0.21	14.74	8.13	11.50	12.60	26.22	3.88	0.05
0.01	201.84	0.54	0.03	0.31	4.16	0.26	15.13	8.57	12.42	13.96	27.40	4.14	0.06
0.01	201.84	0.54	0.03	0.31	3.90	0.16	13.62	7.39	9.89	9.99	20.83	3.08	0.05
0.01	201.84	0.54	0.03	0.31	4.32	0.20	14.77	7.67	10.98	10.52	24.51	3.50	0.06
0.00	239.78	0.47	0.03	0.43	4.22	0.17	15.70	7.43	10.82	11.84	26.07	3.79	0.04
0.00	239.78	0.47	0.03	0.43	4.24	0.19	13.62	7.90	10.48	10.48	22.62	3.31	0.06
0.00	239.78	0.47	0.03	0.43	4.48	0.16	14.54	8.11	10.01	12.06	22.17	3.42	0.05
0.00	239.78	0.47	0.03	0.43	3.14	0.09	10.79	5.36	7.49	5.33	11.69	1.70	0.05
0.00	239.78	0.47	0.03	0.43	4.20	0.16	15.50	6.89	10.29	9.88	22.97	3.29	0.05
0.00	239.78	0.47	0.03	0.43	4.26	0.20	13.54	7.42	10.75	9.68	22.09	3.18	0.06
0.00	239.78	0.47	0.03	0.43	4.31	0.14	13.50	7.35	10.23	9.39	19.97	2.94	0.05
0.00	239.78	0.47	0.03	0.43	4.45	0.19	15.08	7.71	10.87	11.37	24.93	3.63	0.05
0.00	239.78	0.47	0.03	0.43	4.62	0.19	15.28	7.78	10.86	12.69	26.74	3.94	0.05
0.00	239.78	0.47	0.03	0.43	4.35	0.23	13.90	7.27	10.33	10.41	23.41	3.38	0.07
0.00	239.78	0.47	0.03	0.43	4.83	0.28	16.40	7.73	11.83	12.11	27.42	3.95	0.07
0.00	239.78	0.47	0.03	0.43	3.87	0.12	11.63	6.42	9.45	7.26	15.11	2.24	0.05
0.00	239.78	0.47	0.03	0.43	4.01	0.17	12.31	7.35	9.55	10.40	18.77	3.29	0.05
0.00	239.78	0.47	0.03	0.43	4.37	0.15	13.74	7.87	10.52	11.74	24.14	3.29	0.04
0.00	239.78	0.47	0.03	0.43	4.30	0.21	16.26	7.21	10.95	11.49	24.28	3.58	0.06
0.00	239.78	0.47	0.03	0.43	3.87	0.15	14.21	6.93	10.00	8.26	18.99	2.73	0.06
0.00	239.78	0.47	0.03	0.43	4.50	0.28	16.82	8.16	12.38	12.47	30.13	4.26	0.07
0.01	224.71	0.57	0.03	0.34	4.09	0.15	14.09	6.55	10.41	8.54	21.59	3.01	0.05

0.01	224.71	0.57	0.03	0.34	4.36	0.23	14.69	7.48	11.14	11.33	25.27	3.53	0.07
0.01	224.71	0.57	0.03	0.34	4.45	0.18	14.56	7.40	10.30	10.07	31.50	3.15	0.06
0.01	224.71	0.57	0.03	0.34	4.68	0.23	12.99	7.02	11.01	10.02	23.24	3.33	0.07
0.01	224.71	0.57	0.03	0.34	4.87	0.26	14.89	8.14	11.60	12.76	27.26	4.00	0.07
0.01	224.71	0.57	0.03	0.34	4.55	0.24	15.77	7.79	11.36	10.95	25.00	3.60	0.07
0.01	224.71	0.57	0.03	0.34	4.27	0.22	15.82	7.94	12.24	12.32	28.68	4.10	0.05
0.01	224.71	0.57	0.03	0.34	4.28	0.17	12.85	7.42	11.33	10.60	23.94	3.45	0.05
0.01	224.71	0.57	0.03	0.34	4.73	0.20	14.25	7.78	10.18	10.13	22.50	3.26	0.06
0.01	224.71	0.57	0.03	0.34	4.42	0.19	14.70	7.73	11.08	10.49	25.03	3.55	0.06
0.05	301.97	0.52	0.04	0.30	4.14	0.20	14.57	7.21	10.72	9.78	22.30	3.21	0.06
0.05	301.97	0.52	0.04	0.30	4.18	0.23	13.70	7.88	11.29	12.09	25.20	3.73	0.06
0.05	301.97	0.52	0.04	0.30	4.44	0.26	15.23	7.24	10.88	11.45	24.35	3.58	0.07
0.05	301.97	0.52	0.04	0.30	5.10	0.17	14.88	7.75	11.26	10.88	23.67	3.46	0.05
0.05	301.97	0.52	0.04	0.30	4.26	0.18	13.40	6.92	9.75	8.43	20.24	2.87	0.06
0.05	301.97	0.52	0.04	0.30	4.16	0.17	14.71	7.03	11.05	10.17	23.64	3.38	0.05
0.05	301.97	0.52	0.04	0.30	4.39	0.23	14.60	7.72	11.03	11.54	25.05	3.66	0.06
0.05	301.97	0.52	0.04	0.30	4.22	0.19	16.34	6.71	10.19	9.74	22.00	3.17	0.06
0.05	301.97	0.52	0.04	0.30	4.63	0.21	14.45	8.11	11.76	12.13	26.17	3.83	0.06
0.05	301.97	0.52	0.04	0.30	3.81	0.12	12.92	6.48	9.18	7.20	16.87	2.41	0.05
0.05	301.97	0.52	0.04	0.30	3.89	0.19	14.68	7.17	10.66	9.86	21.70	3.16	0.06
0.05	301.97	0.52	0.04	0.30	4.61	0.20	14.85	6.69	10.48	9.45	22.39	3.18	0.06
0.05	301.97	0.52	0.04	0.30	4.16	0.29	14.52	7.48	10.62	10.63	21.76	3.24	0.09
0.05	301.97	0.52	0.04	0.30	4.63	0.23	14.80	8.28	12.26	12.59	29.17	4.18	0.06
0.05	301.97	0.52	0.04	0.30	4.36	0.20	15.06	7.06	10.28	9.47	20.77	3.02	0.07
0.05	281.23	0.66	0.03	0.14	4.25	0.15	14.94	7.55	10.37	10.59	21.71	3.23	0.05
0.05	281.23	0.66	0.03	0.14	4.55	0.22	15.04	7.50	11.45	10.94	24.50	3.54	0.06
0.05	281.23	0.66	0.03	0.14	4.00	0.18	14.35	7.38	9.97	10.18	22.22	3.24	0.06
0.05	281.23	0.66	0.03	0.14	4.02	0.16	12.58	6.76	9.18	8.35	18.02	2.64	0.06
0.05	281.23	0.66	0.03	0.14	3.94	0.16	13.57	7.29	10.34	9.10	22.50	3.16	0.05
0.05	281.23	0.66	0.03	0.14	4.84	0.19	13.21	7.08	10.78	10.52	22.87	3.34	0.06
0.05	281.23	0.66	0.03	0.14	4.32	0.17	14.05	7.90	11.12	11.33	25.54	3.69	0.05
0.05	281.23	0.66	0.03	0.14	4.26	0.19	14.01	7.10	10.07	9.29	21.87	3.12	0.06
0.05	281.23	0.66	0.03	0.14	3.88	0.16	13.19	7.22	10.13	9.17	19.83	2.90	0.06
0.05	281.23	0.66	0.03	0.14	4.59	0.21	13.94	8.05	11.78	11.98	26.57	3.86	0.05
0.05	281.23	0.66	0.03	0.14	3.77	0.19	13.42	7.17	10.27	9.19	21.12	3.03	0.06
0.05	281.23	0.66	0.03	0.14	4.39	0.22	14.41	7.13	10.37	9.47	22.24	3.18	0.07
0.05	281.23	0.66	0.03	0.14	4.24	0.18	15.36	7.40	10.40	10.84	21.70	3.13	0.06
0.05	281.23	0.66	0.03	0.14	4.06	0.16	12.67	6.82	9.75	8.06	16.98	2.50	0.06
0.05	281.23	0.66	0.03	0.14	3.83	0.13	12.64	7.01	10.44	8.73	20.73	2.95	0.04
0.05	281.23	0.66	0.03	0.14	4.55	0.24	13.08	7.19	10.80	9.53	22.72	3.23	0.07
0.05	281.23	0.66	0.03	0.14	3.61	0.15	12.39	6.53	9.61	7.83	17.76	2.56	0.06

Table S3. The three final predictors for the landscape and climate design analyses (note: field border and percentage SNH were collapsed into the first PC axis and used as a predictor variable) and the other three morphological and three environmental predictor variables analyzed (mean ± standard error) for the 304 *B. terrestris* across 19 sites, presented as Site ID, simple (S) or complex (C) landscape type, temperature pair and region (i.e. 1S_5_1).

Site ID	Landscape and climate design variables				Other morphological and environmental variables					
	Latitude	% SNH	Field borders	MaxTemp Warmest Month	ITD	Body length	Wing loading	Mean Annual Precip	% agricultural land cover	% urban land cover
**1S_5_1**	55.49	0.40	244.77	20.94	4.08± 0.15	14.05± 0.31	0.06± 0.00	555.98	76.98	8.63
**2S_1_1**	55.44	0.50	229.94	20.90	4.25± 0.13	14.20± 0.26	0.06± 0.00	670.34	85.26	4.31
**3S_6_1**	55.63	0.58	262.34	21.16	3.80± 0.07	13.51± 0.32	0.05± 0.00	657.66	89.21	4.36
**4C_4_1**	55.48	4.65	294.62	21.14	3.83± 0.11	13.47± 0.35	0.05± 0.00	663.38	80.22	4.33
**5C_5_1**	55.72	6.40	302.24	20.52	4.19± 0.13	14.61± 0.22	0.06± 0.00	787.44	44.95	3.47
**6C_3_1**	55.59	5.58	399.90	21.13	4.20± 0.08	14.24± 0.26	0.06± 0.00	697.72	80.07	4.60
**7C_1_1**	55.53	5.15	344.02	20.85	4.67± 0.10	14.73± 0.16	0.06± 0.00	635.49	80.37	5.03
**8C_7_2**	58.17	5.72	318.01	20.55	4.39± 0.10	14.45± 0.38	0.06± 0.00	797.56	43.83	3.59
**9S_7_2**	58.57	0.56	191.41	20.73	4.36± 0.13	14.07± 0.30	0.06± 0.00	810.28	40.83	3.11
**10S_8_2**	58.45	0.35	237.41	21.11	4.45± 0.11	14.49± 0.27	0.06± 0.00	626.41	49.62	2.90
**11C_8_2**	58.59	6.92	306.52	21.38	4.46± 0.10	14.68± 0.22	0.06± 0.00	623.43	48.86	3.35
**12C_9_2**	58.27	6.61	332.75	20.65	4.10± 0.09	13.98± 0.21	0.06± 0.00	671.58	44.37	5.38
**13S_9_2**	58.57	0.58	215.85	21.36	4.06± 0.08	13.86± 0.42	0.05± 0.00	590.07	60.13	3.14
**14C_11_3**	59.07	6.68	283.21	21.68	4.21± 0.11	14.29± 0.31	0.06± 0.00	697.70	49.13	2.93
**15S_11_3**	59.17	0.58	201.84	22.18	4.26± 0.09	14.79± 0.29	0.06± 0.00	651.23	54.25	3.19
**16S_12_3**	59.44	0.43	239.78	22.09	4.23± 0.09	14.28± 0.41	0.05± 0.00	644.58	46.52	3.20
**17S_13_3**	59.78	0.66	224.71	22.12	4.47± 0.08	14.46± 0.31	0.06± 0.00	607.94	56.73	3.46
**18C_12_3**	59.67	4.55	301.96	22.20	4.33± 0.08	14.58± 0.21	0.06± 0.00	568.06	52.15	3.75
**19C_13_3**	59.69	4.63	281.23	21.74	4.18± 0.08	13.70± 0.22	0.06± 0.00	555.37	66.50	2.82

Note: the variable SeasonPrecip was not used (see Methods) and therefore not included in the table.

Table S4. Variance inflation factor (VIF) of the landscape and climate study design variables and the other environmental and morphological predictor variables used to run the two RDA models. Sampling design variables: Latitude, MaxTempWarmestMonth= the maximum temperature of the warmest month, °C and PC1_SNH_FB= the first PCA axis on percentage of SNH land cover and length of uncultivated field borders. Environmental variables: MeanAnnualPrecip= mean annual precipitation (mm), SeasonPrecip= precipitation seasonality, the difference between the driest and wettest month in %, % agriculture= the percentage of agricultural land cover and % urban area= the percentage of urban land cover. Morphological variables: ITD= inter tegular distance (mm), Body length= length of the body (mm) and Wing loading= the body weight in grams divided by the total wing area in cm^2 (g/cm^2).

**Landscape and climate study design variables**	
	**VIF**
Latitude	1.66
MaxTempWarmestMonth, °C	1.69
PC1_SNH_FB	1.06

**Other environmental and morphological variables**			
	**VIF**	**Reduced dataset**	**VIF**
MeanAnnualPrecip, mm	4.16	MeanAnnualPrecip, mm	1.16
SeasonPrecip, mm	6.56	% agricultural land cover	1.44
% agricultural land cover	2.70	% urban land cover	1.30
% urban land cover	3.68	ITD, mm	1.58
ITD, mm	1.58	Body length, mm	1.55
Body length, mm	1.55	Wing loading, g/cm^2	1.07
Wing loading, g/cm^2	1.06		

Table S5. Estimated allelic richness (A_R), expected heterozygosity (H_E), observed heterozygosity (H_O), the inbreeding coefficient (F_{IS}), and Tajima's D with and without linkage disequilibrium (LD) filter per site for the 304 individuals are presented. * Indicates statistically significant inbreeding coefficient within a site. Site is presented as Site ID, simple (S) or complex (C) landscape type, temperature pair and region (i.e. 1S_5_1).

Site	N	A_R	H_O	H_E	F_{IS}	Tajima's D	Tajima's D (no LD filter)
**1S_5_1**	16	1.482	0.133	0.143	0.050*	-0.308	-0.313
**2S_1_1**	16	1.478	0.129	0.142	0.064*	-0.296	-0.301
**3S_6_1**	17	1.479	0.126	0.143	0.081*	-0.292	-0.295
**4C_4_1**	17	1.481	0.124	0.143	0.095*	-0.282	-0.286
**5C_5_1**	17	1.485	0.136	0.143	0.037*	-0.297	-0.301
**6C_3_1**	17	1.480	0.133	0.142	0.047*	-0.296	-0.298
**7C_1_1**	17	1.481	0.134	0.142	0.040*	-0.310	-0.310
**8C_7_2**	17	1.486	0.135	0.145	0.042*	-0.302	-0.306
**9S_7_2**	16	1.489	0.137	0.145	0.041*	-0.285	-0.287
**10S_8_2**	17	1.483	0.132	0.144	0.056*	-0.290	-0.293
**11C_8_2**	14	1.486	0.135	0.143	0.042*	-0.291	-0.293
**12C_9_2**	17	1.486	0.134	0.144	0.043*	-0.311	-0.313
**13S_9_2**	17	1.488	0.127	0.144	0.084*	-0.292	-0.297
**14C_11_3**	17	1.485	0.137	0.144	0.042*	-0.266	-0.269
**15S_11_3**	13	1.491	0.137	0.145	0.037*	-0.298	-0.301
**16S_12_3**	17	1.486	0.134	0.145	0.058*	-0.262	-0.265
**17S_13_3**	10	1.481	0.132	0.144	0.055*	-0.262	-0.266
**18C_12_3**	15	1.482	0.133	0.143	0.050*	-0.289	-0.291
**19C_13_3**	17	1.490	0.136	0.144	0.042*	-0.286	-0.290
**Mean**	16	1.484	0.132	0.144	0.053	-	

Table S6. Matrix representing bootstrapping (10000 permutations) over loci of pairwise F_{ST}. Upper limit (95%) is above the diagonal and the lower limit (5%) is below the diagonal. Significant pairwise F_{ST} values are shown in bold.

	1	2	3	4	5	6	7	8	9	10	11	12	13	14	15	16	17	18	19
**1**	NA	0.0007	0.0019	0.0005	0.0009	−0.0011	0.0024	**0.0041**	**0.0044**	0.0027	**0.0038**	0.0022	0.0032	**0.0043**	0.0036	0.0032	0.0040	**0.0065**	**0.0058**
**2**	-0.0026	NA	0.0018	0.0025	0.0008	−0.0004	0.0025	0.0026	0.0021	0.0015	0.0020	0.0020	0.0033	0.0029	0.0036	0.0024	0.0025	0.0034	0.0050
**3**	-0.0012	-0.0013	NA	0.0027	0.0006	0.0004	0.0025	0.0045	0.0037	0.0020	**0.0036**	**0.0039**	**0.0033**	**0.0064**	**0.0040**	**0.0042**	0.0041	**0.0047**	**0.0048**
**4**	-0.0027	-0.0011	-0.0007	NA	0.0010	0.0012	0.0023	0.0031	0.0033	**0.0041**	0.0018	0.0018	0.0023	**0.0039**	0.0013	**0.0034**	0.0038	0.0032	**0.0046**
**5**	-0.0022	-0.0024	-0.0024	-0.0023	NA	0.0001	0.0009	**0.0035**	0.0033	**0.0036**	0.0025	0.0020	0.0022	**0.0050**	0.0034	**0.0037**	0.0034	**0.0039**	**0.0033**
**6**	-0.0040	-0.0033	-0.0026	-0.0020	-0.0026	NA	0.0013	**0.0033**	0.0003	0.0028	0.0026	0.0010	0.0010	**0.0036**	0.0033	0.0025	0.0036	**0.0041**	**0.0042**
**7**	-0.0006	-0.0007	-0.0009	-0.0008	-0.0023	−0.0018	NA	**0.0056**	**0.0036**	**0.0039**	**0.0043**	**0.0038**	**0.0040**	**0.0036**	**0.0045**	**0.0060**	**0.0060**	**0.0046**	**0.0049**
**8**	**0.0008**	-0.0006	**0.0009**	-0.0001	**0.0003**	**0.0002**	**0.0021**	NA	**0.0043**	**0.0047**	0.0018	0.0027	0.0028	**0.0039**	0.0036	**0.0044**	0.0033	0.0028	**0.0045**
**9**	**0.0010**	-0.0013	**0.0002**	-0.0002	0.0000	−0.0026	**0.0002**	**0.0010**	NA	0.0030	0.0028	0.0029	0.0032	**0.0039**	0.0028	**0.0044**	0.0035	0.0028	**0.0034**
**10**	-0.0004	-0.0019	-0.0011	**0.0006**	**0.0004**	−0.0005	**0.0005**	**0.0011**	-0.0004	NA	0.0018	0.0025	0.0024	**0.0040**	0.0021	0.0028	0.0031	0.0035	**0.0040**
**11**	**0.0002**	-0.0016	**0.0001**	-0.0016	-0.0008	−0.0008	**0.0005**	−0.0016	-0.0006	−0.0016	NA	0.0019	0.0023	0.0029	0.0001	**0.0037**	0.0015	0.0020	0.0025
**12**	-0.0009	-0.0013	**0.0005**	-0.0015	-0.0011	−0.0022	**0.0005**	−0.0004	-0.0005	−0.0005	−0.0012	NA	0.0015	0.0031	0.0012	0.0021	0.0019	**0.0036**	0.0033
**13**	-0.0001	0.0000	**0.0000**	-0.0011	-0.0010	−0.0020	**0.0007**	−0.0004	-0.0001	−0.0007	−0.0012	−0.0019	NA	**0.0038**	0.0026	**0.0034**	0.0033	0.0028	**0.0035**
**14**	**0.0009**	-0.0003	**0.0028**	**0.0007**	**0.0017**	**0.0005**	**0.0004**	**0.0006**	**0.0006**	**0.0007**	−0.0005	−0.0002	**0.0004**	NA	0.0032	0.0026	0.0040	0.0025	**0.0036**
**15**	-0.0003	-0.0003	**0.0002**	-0.0021	-0.0001	−0.0005	**0.0007**	−0.0001	-0.0011	−0.0016	−0.0037	−0.0022	-0.0011	−0.0006	NA	0.0033	0.0040	0.0033	0.0023
**16**	-0.0001	-0.0009	**0.0007**	**0.0000**	**0.0006**	−0.0007	**0.0025**	**0.0011**	**0.0008**	−0.0004	**0.0002**	−0.0001	**0.0003**	−0.0004	−0.0005	NA	0.0044	0.0013	**0.0036**
**17**	-0.0004	-0.0018	-0.0003	-0.0006	-0.0009	−0.0006	**0.0018**	−0.0010	-0.0006	−0.0012	−0.0029	−0.0024	-0.0012	−0.0002	−0.0007	−0.0001	NA	0.0032	0.0016
**18**	**0.0028**	-0.0001	**0.0012**	-0.0004	**0.0002**	**0.0006**	**0.0009**	−0.0005	-0.0006	−0.0000	−0.0017	**0.0001**	-0.0008	−0.0009	−0.0007	−0.0018	−0.0001	NA	0.0027
**19**	**0.0022**	**0.0014**	**0.0014**	**0.0010**	**0.0001**	**0.0009**	**0.0014**	**0.0010**	**0.0001**	**0.0007**	−0.0001	−0.0001	**0.0000**	**0.0003**	−0.0015	**0.0004**	−0.0025	−0.0007	NA

Table S7. Spearman rank correlations test of full time series and subset (2000-2023) time series. Significant values are shown in bold.

Metric	Time period	ρ (Spearman)	p-value
Mean latitude	1970–2023	–0.09	0.55
90th percentile latitude	1970–2023	0.67	**<0.001**
Mean latitude	2000–2023	0.48	**0.018**
90th percentile latitude	2000–2023	0.48	**0.018**

Table S8. Full redundancy analysis (RDA) model for the landscape and climate sampling study predictors and significant axes used for outlier detection.

	Predictor variables				
	Df	Variance	F	Pr(>F)	R^2
	3	75.3	1.1367	0.001	0.001
**One axis**	**Df**	**Variance**	**F**	**Pr(>F)**	R^2
RDA1	1	30.5	1.3823	0.001	-

Table S9. Full redundancy analysis (RDA) model for the other environmental and morphological predictors and significant axes used for outlier detection.

	Predictor variables				
	Df	Variance	F	Pr(>F)	R^2
	6	139.1	1.0492	0.001	0.0001
**One axis**	**Df**	**Variance**	**F**	**Pr(>F)**	**R^2**
RDA1	1	28.9	1.3083	0.001	-

Table S10. Overlapping outlier SNPs between the two RDA analyses for RDA axis 1. The gene name, the predictors for the simple and complex landscape and temperature study design RDA analysis, and the predictors for the other environmental and morphological RDA analysis are presented.

Predictor landscape and climate design	Predictor env. and morph.	Gene name
Latitude	% urban land cover	uncharacterized
Latitude	% agricultural land cover	B-cell receptor CD22
Latitude	% urban land cover	transcription factor collier
Latitude	% urban land cover	latrophilin Cirl
Latitude	% agricultural land cover	uncharacterized
Latitude	% agricultural land cover	semaphorin-1A
Latitude	% agricultural land cover	dipeptidase 1
Latitude	% agricultural land cover	odorant receptor 82a-like
MaxTempWarmestMonth	% agricultural land cover	TWiK family of potassium channels protein 18
Latitude	Body length mm	hemicentin-1
Latitude	% urban land cover	myocardin-related transcription factor B
Latitude	% urban land cover	protein artichoke
Latitude	% agricultural land cover	phosphatidylinositol-glycan biosynthesis class X protein
Latitude	% agricultural land cover	longitudinals lacking protein
Latitude	MeanAnnualPrecip	peripheral plasma membrane protein CASK
Latitude	% urban land cover	CKLF-like MARVEL transmembrane domain-containing protein 4
Latitude	% urban land cover	frizzled-2
Latitude	% agricultural land cover	disks large 1 tumor suppressor protein
Latitude	% urban land cover	homeobox protein unc-42
Latitude	MeanAnnualPrecip	zinc finger protein rotund
Latitude	% agricultural land cover	uncharacterized
Latitude	% agricultural land cover	dendritic arbor reduction protein 1
Latitude	% agricultural land cover	phosphatidylinositol-glycan biosynthesis class X protein
Latitude	% agricultural land cover	uncharacterized
Latitude	% agricultural land cover	cAMP-specific 3',5'-cyclic phosphodiesterase
Latitude	% agricultural land cover	guanine nucleotide-binding protein G(s) subunit alpha

Table S11. The 49 candidate outlier SNPs identified for RDA1 in the landscape and climate study design RDA analysis. The RefSeq (chromosome), Loci ID, SNP position, gene name, predictor variables and VEP consequence is presented.

RefSeq (chr)	Loci ID DarT	SNP position	Predictor	LOC ID	Gene name	VEP
0	82202452-33-A/G	0	Latitude	LOC105665892	uncharacterized	-
B01	82203083-37-T/C	1700556	Latitude	LOC00651512	B-cell receptor CD22	intron
B01	82213829-5-T/G	2994264	Latitude	LOC100649283	latrophilin Cirl	intron
B01	82201644-12-C/G	4290894	Latitude	LOC100645818	cysteine-rich motor neuron 1 protein	intron
B01	82203219-24-C/T	6735884	Latitude	LOC100644085	uncharacterized	intron
B01	82198968-40-C/T	7223240	Latitude	LOC100649436	leucine-rich repeat and calponin homology domain-containing protein	Downstream gene, intron
B01	82210316-67-C/G	9141189	Latitude	LOC100650834	transcription factor collier	5 prime UTR, non coding transcript exon
B01	82202130-11-A/G	13782177	Latitude	LOC100650993	uncharacterized	intron
B01	82198141-49-G/C	16846494	Latitude	LOC100645821	uncharacterized	intron
B02	82198779-34-C/T	2055348	MaxTemp Warmest Month	LOC100650835	uncharacterized	intron
B02	82203019-62-A/C	2911546	Latitude	LOC100647567	semaphorin-1A	intron
B02	82211541-41-C/T	6916767	Latitude	LOC100649987	dipeptidase 1	intron
B02	82202011-23-G/A	8321631	Latitude	LOC100647925	irregular chiasm C-roughest protein	intron
B02	82203975-27-T/C	10197053	Latitude	LOC100646456	odorant receptor 82a-like	Downstream gene, synonymous, upstream gene variant
B03	82199998-8-C/T	1433477	Latitude	LOC100651751	sex-regulated protein janus-A	Intron, non coding transcript
B03	82199998-8-C/T	1433477	Latitude	LOC100650954	phosrestin-2	Intron, no coding transcript
B03	82203208-32-G/A	5362122	MaxTemp Warmest Month	LOC100648390	TWiK family of potassium channels protein 18	Intron, non coding transcript
B05	82202568-35-G/A	8054397	Latitude	LOC100647333	sodium/potassium-transporting ATPase subunit alpha	intron
B05	82198536-24-T/C	9165266	Latitude	LOC100650879	hemicentin-1	Downstream gene
B07	82200381-28-C/T	1746104	Latitude	LOC100650810	prickle planar cell polarity protein 3	Downstream gene
B07	82203276-66-G/A	8497982	Latitude	LOC100649623	myocardin-related transcription factor B	intron
B08	82199021-24-G/A	6420486	Latitude	LOC100650649	protein artichoke	intron

B09	82203875-29-C/A	289418	Latitude	LOC100647976	phosphatidylinositol-glycan biosynthesis class X protein	5 prime UTR, intron, upstream gene
B09	82212871-15-C/T	1424836	Latitude	LOC100649963	longitudinals lacking protein	intron
B09	82202826-33-G/C	5566344	MaxTemp Warmest Month	LOC100645564	peripheral plasma membrane protein CASK	intron
B09	82202211-22-C/A	9094003	Latitude	LOC100642694	CKLF-like MARVEL transmembrane domain-containing protein 4	Upstream gene, downstrea gene
B09	82200316-55-C/T	13577108	Latitude	LOC100651492	retinoic acid receptor RXR-alpha-B	intron
B09	82198855-57-A/G	15261982	Latitude	LOC100651687	protein phosphatase Slingshot	intron
B10	82202797-12-T/C	1165331	Latitude	LOC100643816	frizzled-2	intron
B10	82196127-61-C/T	12406534	Latitude	LOC100645644	tetraspanin-5	intron
B11	82203126-36-C/T	15027633	Latitude	LOC100644734	single Ig IL-1-related receptor	intron
B12	82197183-66-G/A	818706	Latitude	LOC100643741	mucin-5AC	Downstream gene, intron
B12	82203717-57-C/T	1077156	Latitude	LOC100643982	disks large 1 tumor suppressor protein	intron
B12	82201844-59-C/T	3892865	Latitude	LOC100650779	LIM domain transcription factor LMO4.1	intron
B12	82201044-45-G/T	5405283	Latitude	LOC100642737	agrin	intron
B13	82202572-58-G/A	6260158	MaxTemp Warmest Month	LOC100651851	transmembrane protein 132C	missense, downstream gene
B13	82203897-39-T/C	7632750	Latitude	LOC100649943	homeobox protein unc-42	intron
B14	82198206-52-T/C	4801627	Latitude	LOC100643342	zinc finger protein rotund	intron
B14	82214293-10-C/T	5559617	Latitude	LOC100652294	uncharacterized	intron
B14	82204052-52-T/C	8687293	Latitude	LOC100651344	thyrotroph embryonic factor	Upstream gene, 3 prime UTR, upstream gene
B14	82202979-65-C/T	9555991	Latitude	LOC100644907	uncharacterized	intron
B15	82202051-5-G/C	4325114	Latitude	LOC100650702	dopamine receptor 2	intron, non coding transcript
B15	100057650-21-C/T	4468226	Latitude	LOC100651542	dendritic arbor reduction protein 1	intron
B15	82203874-7-G/A	7316155	Latitude	LOC100647976	phosphatidylinositol-glycan biosynthesis class X protein	3 prime UTR, ustream gene, intron
B15	82197151-28-C/T	8943460	Latitude	LOC100646518	uncharacterized	intron
B16	82197481-34-G/A	1422636	Latitude	LOC100648035	cAMP-specific 3',5'-cyclic phosphodiesterase	intron
Group Un12	82204496-20-G/C	4714	Latitude	LOC105666726	uncharacterized	-
Group Un154	82203080-44-T/A	545515	Latitude	LOC100642353	guanine nucleotide-binding protein G(s) subunit alpha	-
Group Un981	82197313-46-G/T	690139	Latitude	LOC100651185	alpha-mannosidase 2	-

Table S12. The 52 candidate outlier SNPs identified for RDA1 in the other environmental and morphological RDA analysis. The RefSeq (chromosome), Loci ID, SNP position, gene name, predictor variables, and VEP consequence are presented.

RefSeq (chr)	Loci ID DarT	SNP position	Predictor	LOC ID	Gene name	VEP
0	82202452-33-A/G	0	% urban land cover	LOC105665892	uncharacterized	-
B01	82203083-37-T/C	1700556	% agricultural land cover	LOC100651512	B-cell receptor CD22	intron
B01	82213829-5-T/G	2994264	% urban land cover	LOC100649283	latrophilin Cirl	intron
B01	82210316-67-C/G	9141189	% urban land cover	LOC100650834	transcription factor collier	5 prime UTR, non coding transcript, exon
B01	82198771-57-C/G	1423754 7	% agricultural land cover	LOC100643722	protein eva-1	intron
B01	82198141-49-G/C	1684649 4	% agricultural land cover	LOC100645821	uncharacterized	intron
B02	82198070-11-G/A	2894471	% agricultural land cover	LOC100647567	semaphorin-1A	intron
B02	82203019-62-A/C	2911546	% agricultural land cover	LOC100647567	semaphorin-1A	intron
B02	82211541-41-C/T	6916767	% agricultural land cover	LOC100649987	dipeptidase 1	intron
B02	82203975-27-T/C	1019705 3	% agricultural land cover	LOC100646456	odorant receptor 82a-like	Downstream gene, synonymous , upstream gene variant
B03	82203338-14-C/T	2354786	% agricultural land cover	LOC100648160	protein-tyrosine sulfotransferase	intron
B03	82203208-32-G/A	5362122	% agricultural land cover	LOC100648390	TWiK family of potassium channels protein 18	intron, non coding transcript
B03	82199802-44-C/T	6262181	% agricultural land cover	LOC100652108	proto-oncogene tyrosine-protein kinase receptor Ret	Inton
B04	82201080-37-G/A	2271844	% agricultural land cover	LOC100649725	1-phosphatidylinositol 4,5-bisphosphate phosphodiesterase epsilon-1	intron
B04	82197215-25-G/A	2976649	% agricultural land cover	LOC100645439	uncharacterized	intron
B04	82202192-18-C/T	3358687	% agricultural land cover	LOC100646289	protein scalloped	intron
B04	82211542-34-T/C	4485828	% agricultural land cover	LOC100646738	uncharacterized	intron
B05	82214103-67-G/A	2852904	% agricultural land cover	LOC100650807	uncharacterized	intron
B05	82213063-59-G/C	6067075	% urban land cover	LOC100643887	neuroendocrine convertase 2	intron
B05	82198908-8-C/T	7141298	% agricultural land cover	LOC100645828	dystroglycan 1	intron

B05	82198536-24-T/C	9165266	Body length mm	LOC100650879	hemicentin-1	intron
B07	82197279-41-G/A	2306819	% urban land cover	LOC100647420	armadillo repeat-containing protein 8	Upstream gene, 5 prime UTR, downstream gene
B07	82203276-66-G/A	8497982	% urban land cover	LOC100649623	myocardin-related transcription factor B	intron
B07	82203990-58-C/G	15173652	% agricultural land cover	LOC100645913	glutamate receptor 1	intron
B07	82203483-48-A/G	17433013	% agricultural land cover	LOC100645197	protein couch potato	intron
B08	82199021-24-G/A	6420486	% urban land cover	LOC100650649	protein artichoke	intron
B09	82203875-29-C/A	289418	% agricultural land cover	LOC100647976	phosphatidylinositol-glycan biosynthesis class X protein	5 prime UTR, intron, upstream gene
B09	82204480-42-T/C	294888	% agricultural land cover	LOC100647976	phosphatidylinositol-glycan biosynthesis class X protein	Upstream gene, intron
B09	82212871-15-C/T	1424836	% agricultural land cover	LOC100649963	longitudinals lacking protein	intron
B09	82202826-33-G/C	5566344	MeanAnnualPrecip	LOC100645564	peripheral plasma membrane protein CASK	Intron
B09	82202211-22-C/A	9094003	% urban land cover	LOC100642694	CKLF-like MARVEL transmembrane domain-containing protein 4	Upstream gene, downstream gene, intron
B09	82203520-62-G/A	10239784	% urban land cover	LOC100649264	neuroligin-4, X-linked	intron
B09	82202388-19-G/A	12363558	% agricultural land cover	LOC100645528	pinopsin	intron
B10	82202797-12-T/C	1165331	% urban land cover	LOC100643816	frizzled-2	intron
B10	82212649-55-G/A	3856613	% agricultural land cover	LOC100649066	uncharacterized	intron
B10	82203921-59-G/A	7048490	% urban land cover	LOC100644371	ETS-like protein pointed	Upstream gene, intron
B11	82198862-7-A/T	15598686	% urban land cover	LOC100648761	neurogenic locus protein delta	intron
B12	82203717-57-C/T	1077156	% agricultural land cover	LOC100643982	disks large 1 tumor suppressor protein	intron
B12	82201126-9-G/A	3315325	% agricultural land cover	LOC100651536	follistatin-related protein 5	intron, downstream gene
B12	82199484-47-G/A	7850390	% agricultural land cover	LOC100642295	chondroadherin-like protein	intron
B12	82198832-11-A/G	9429838	% urban land cover	LOC100644494	ankyrin repeat domain-containing protein 6	intron
B13	82203897-39-T/C	7632750	% urban land cover	LOC100649943	homeobox protein unc-42	intron
B14	82202939-65-G/A	593178	MeanAnnualPrecip	LOC100643094	tyrosine-protein phosphatase Lar	intron
B14	82198206-52-T/C	4801627	MeanAnnualPrecip	LOC100643342	zinc finger protein rotund	intron

B14	82202979-65-C/T	9555991	% agricultural land cover	LOC100644907	uncharacterized	intron
B15	100057650-21-C/T	4468226	% agricultural land cover	LOC100651542	dendritic arbor reduction protein 1	intron
B15	82203874-7-G/A	7316155	% agricultural land cover	LOC100647976	phosphatidylinositol-glycan biosynthesis class X protein	3 prime UTR, upstream gene
B15	82197151-28-C/T	8943460	% agricultural land cover	LOC100646518	uncharacterized	intron
B16	82197481-34-G/A	1422636	% agricultural land cover	LOC100648035	cAMP-specific 3',5'-cyclic phosphodiesterase	intron
B16	82202918-58-A/C	2919739	% agricultural land cover	LOC100647481	acetylcholinesterase	intron
Group Un154	82203080-44-T/A	545515	% agricultural land cover	LOC100642353	guanine nucleotide-binding protein G(s) subunit alpha	Non coding transcript, intron
Group Un1054	82196892-29-T/C	742046	% agricultural land cover	LOC100651590	cadherin-87A	intron

Supplementary figures

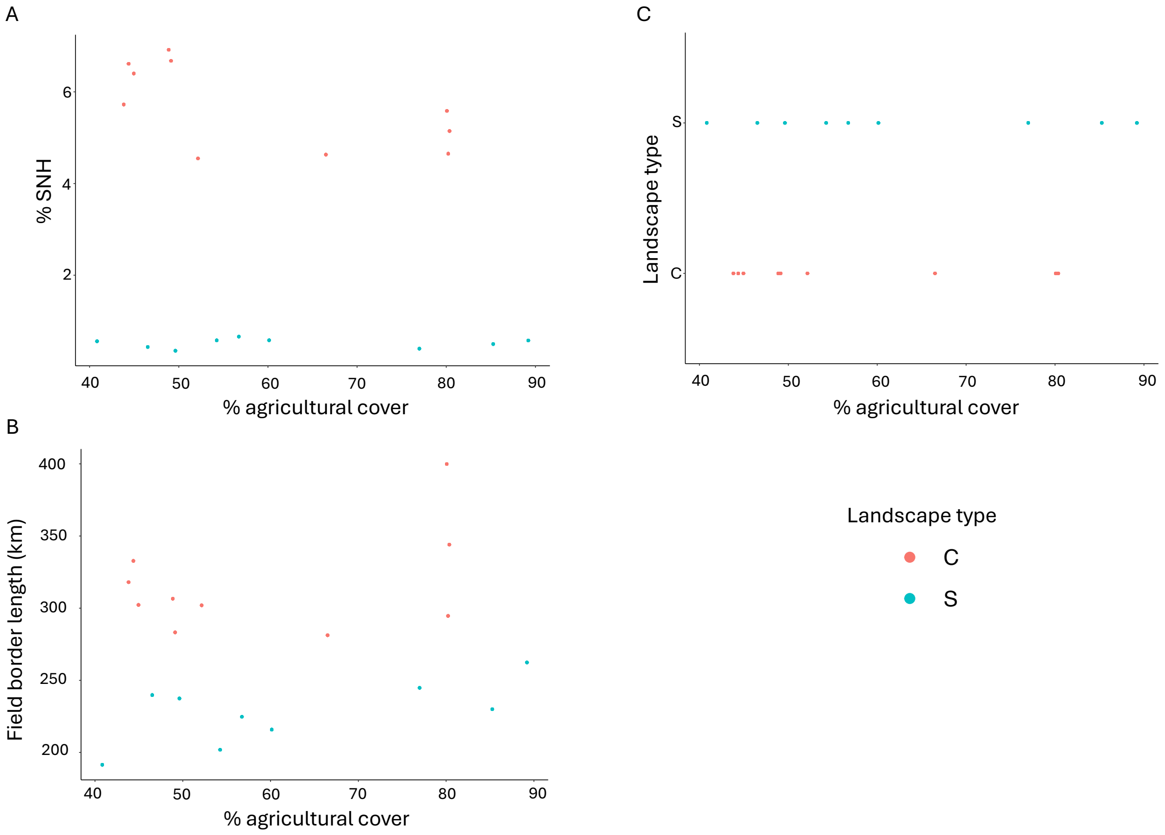

Figure S1. A) the percentage of semi-natural habitat (SNH) and the percentage of agricultural cover, b) the length of uncultivated field borders (km) and percentage of agricultural cover, and c) the percentage of agricultural cover for the simple and complex study sites.

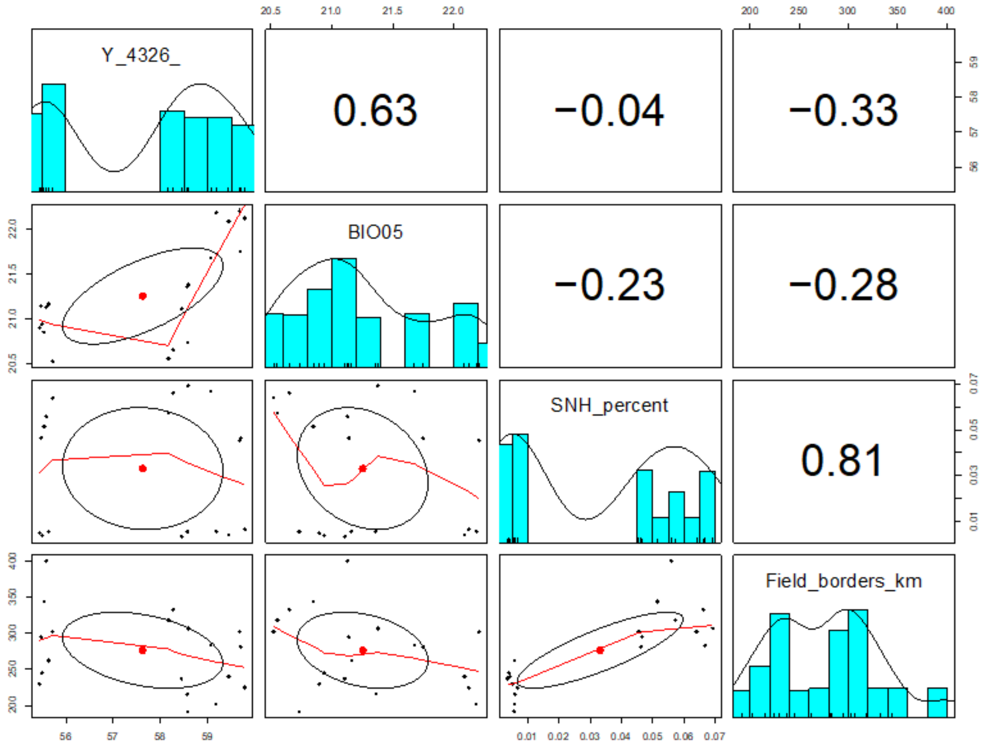

Figure S2. Pearson's correlation matrix for the landscape and climate study design predictor variables. Y_4326= latitude, BIO05= maximum temperature of the warmest month, °C, SNH_percent= percentage of semi-natural habitat, and Field_borders_km= length of uncultivated agricultural field borders, km. SNH_percent and Field_borders_km were collapsed into PC1_SNH_FB.

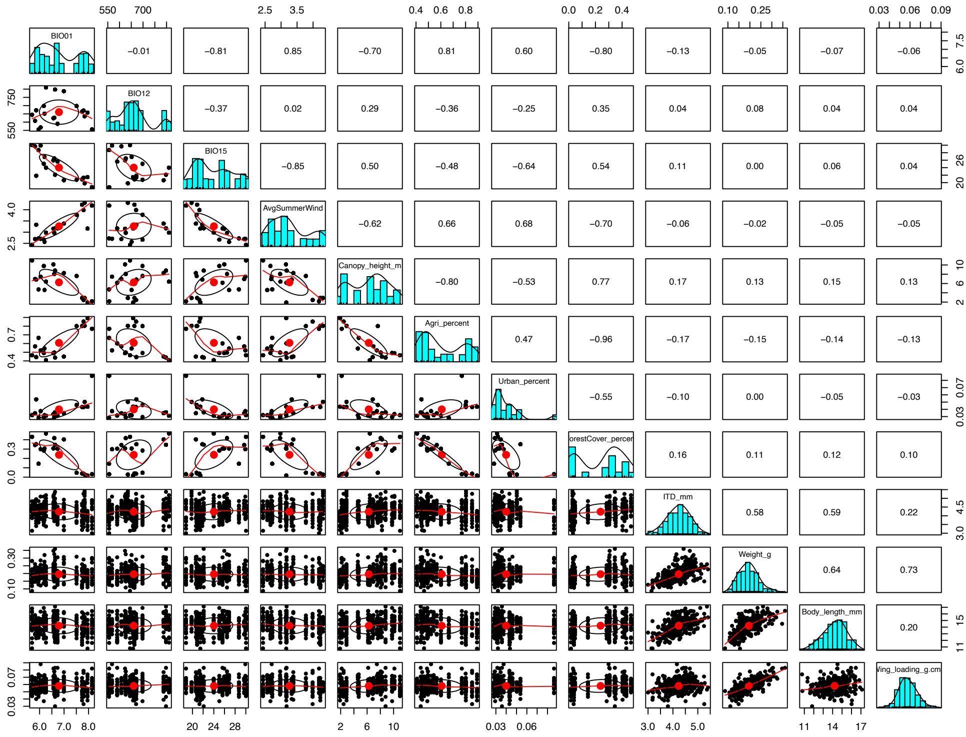

Figure S3. Pearson's correlation matrix for the other environmental and morphological predictor variables. Correlations between variables of r >0.70 were removed. BIO01= MeanAnnualTemp, °C, BIO12= MeanAnnualPrecip, mm, BIO15= SeasonPrecip, Avg_wing_summer= average summer wind, m/s, Canopy_height_m= height of vegetation, Agri_percent= percentage agricultural land cover, Urban_percent= percentage urban land cover, ForestCover_percentage= percentage of forest cover, ITD_mm= ITD in mm, Weight_g= body weight, Body_length_mm= body length and Wing_loading_g.cm2= wing loading g/cm^2.

A

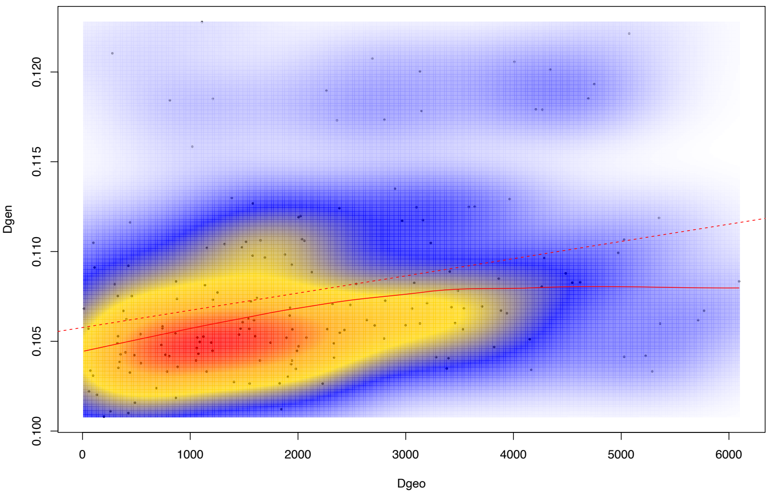

B

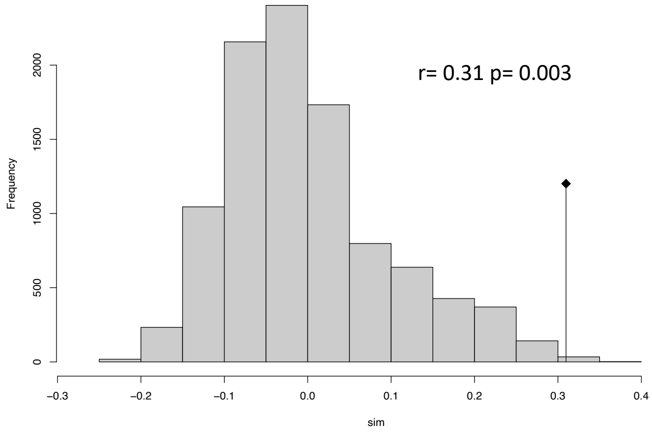

Figure S4. Isolation by Distance (IBD) analysis. a) kernel density estimates of the pairwise correlations between each of the 304 individuals; b) empirical genetic and geographic distance Mantel correlations test (black diamond) plotted over a histogram of 10000 permutations. The simulated p-value of 0.002 indicates significant isolation by distance.

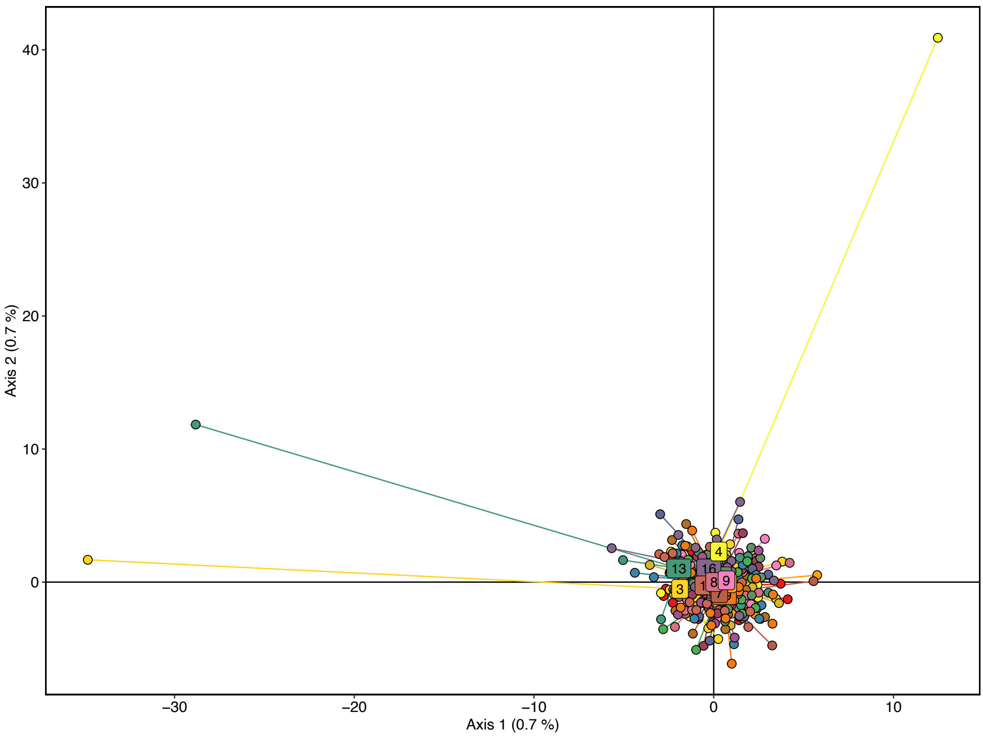

Figure S5. Principal component of Analysis (PCA) of the 304 *B. terrestris* individuals. All individuals from the 19 sites cluster close together except for three outlier individuals. These were removed from the dataset due to missing data or low read depth.

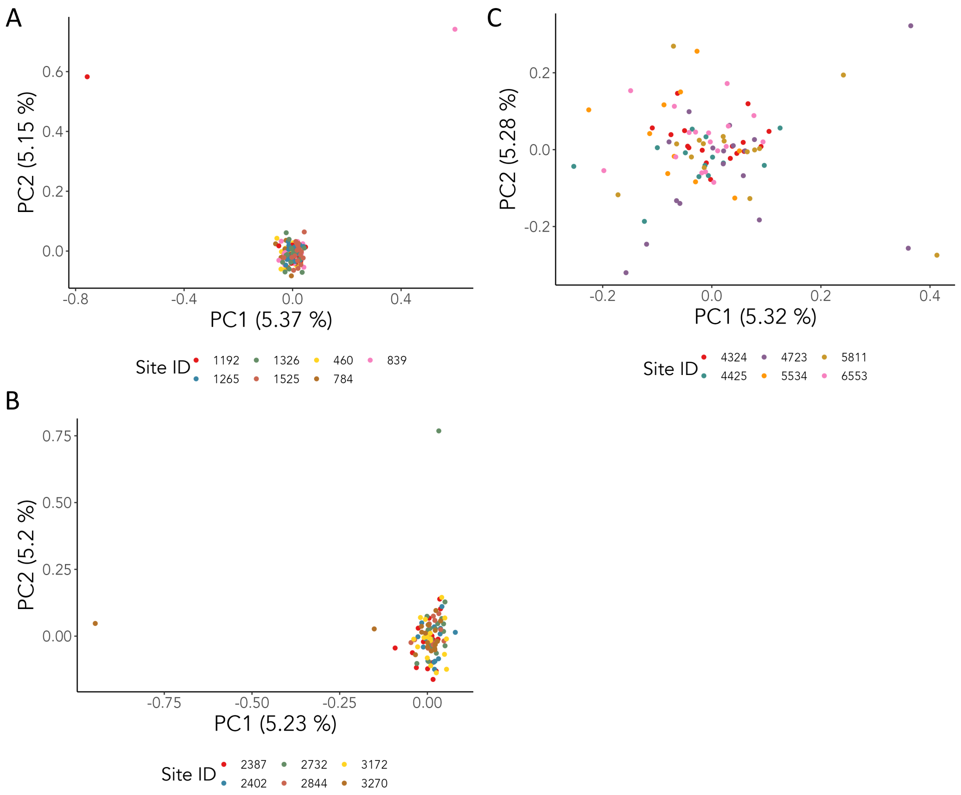

Figure S6. Principal component of Analysis (PCA) showing a) region 1; b) region 2 and c) region 3. The outliers in PCA area 1 and PCA area 2 were removed from the dataset due to low read depth or missing data.

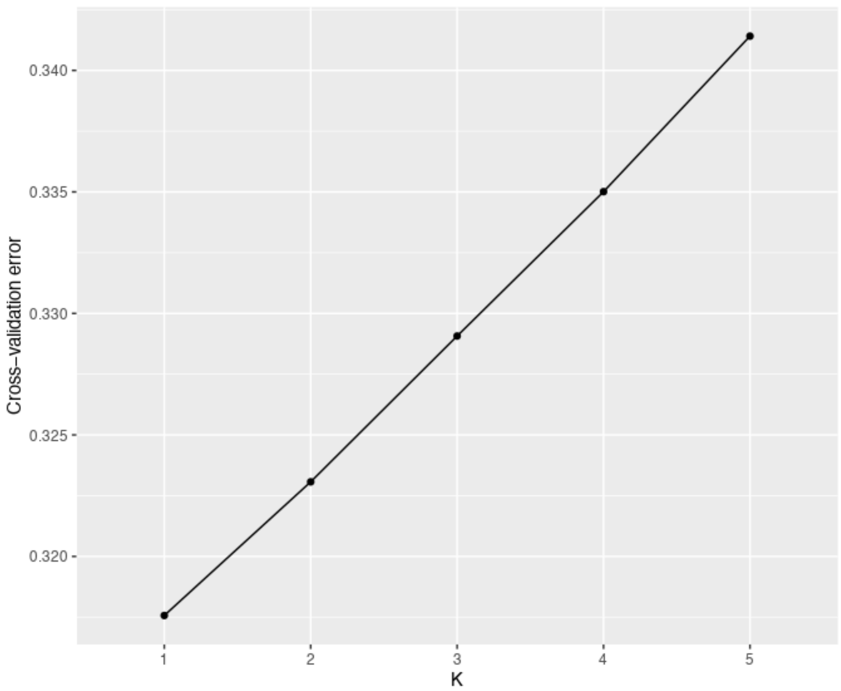

Figure S7. Cross validation (CV) procedure of K= 1-5 in the ADMIXTURE analysis.

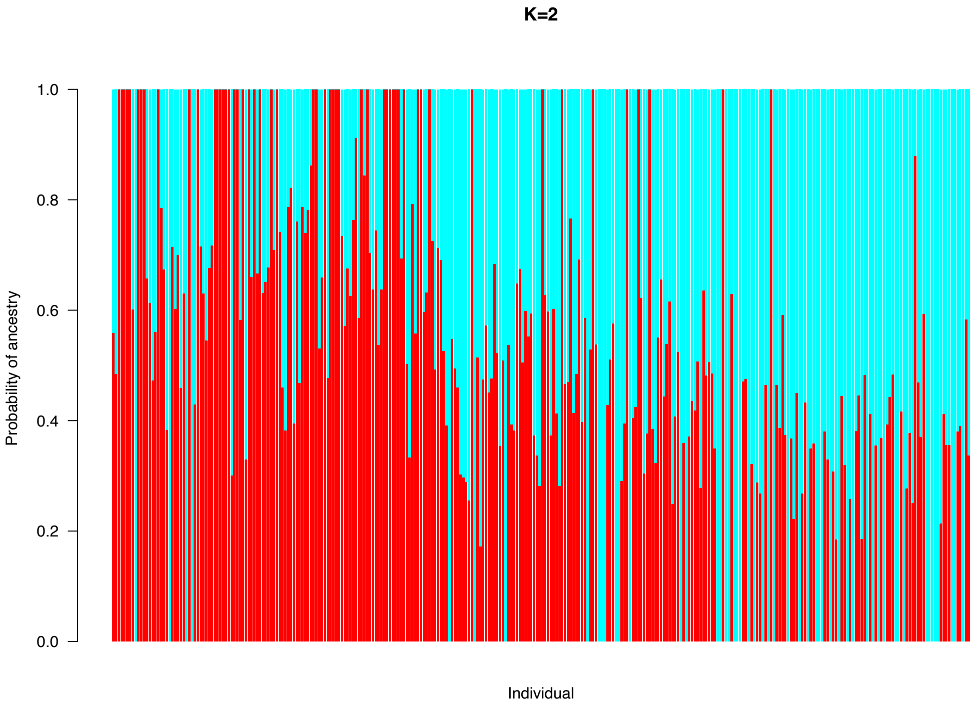

Figure S8. The probability of ancestry to genetic clusters one (red) and two (blue) (K=2), where each bar represents an individual (N=304).

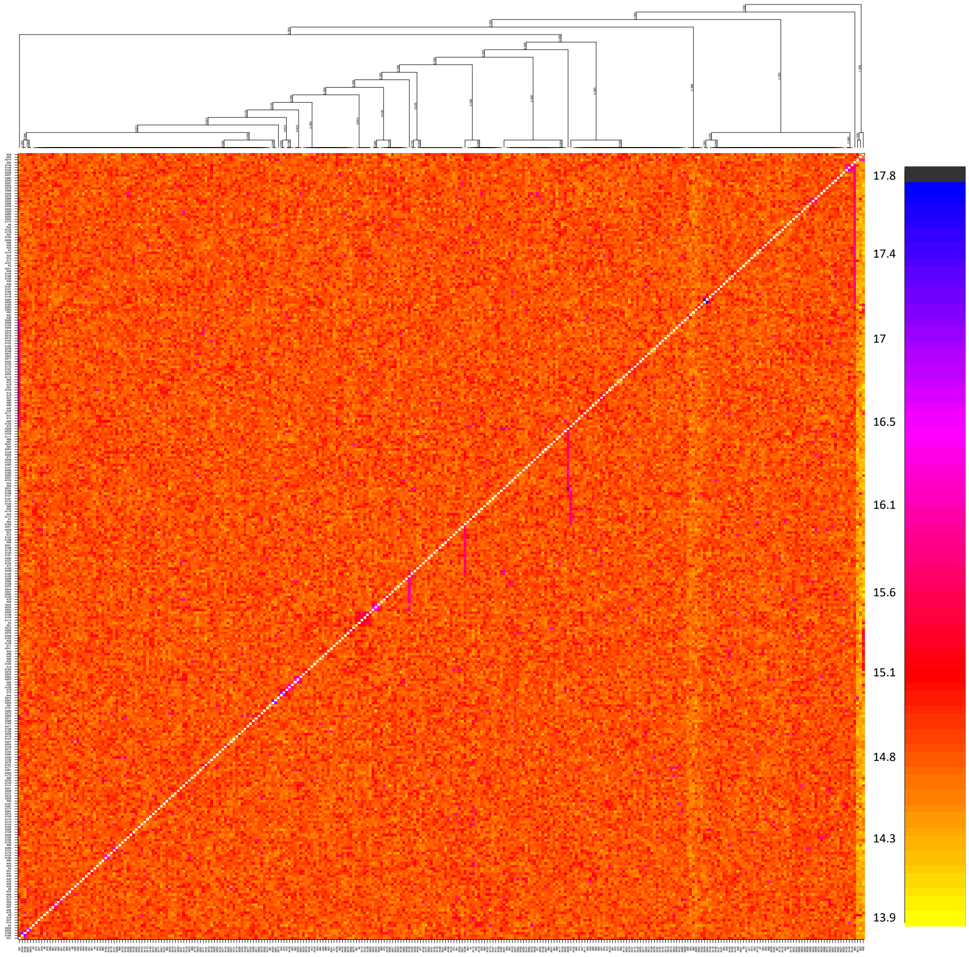

Figure S9. A co-ancestry matrix visualised as a heatmap, generated by fineRADstructure and RADpainter, showing little to nongenetic divergence among the samples. The colour of each cell in the matrix shows the number of expected shared genetic chunks copied from a donor genome (column) to a recipient genome (row). Related individuals (< 0.177), although not full-siblings (1st degree sibling ranges between 0.177– 0.354) are represented by the pink/purple cells. The clustering dendrogram on top of the heatmap forms 29 lineages. Support for the branches on the co- ancestry tree are almost exclusively 0, and only some that ranges between 0.089-1.The individual samples are shown on the X and Y-axis and are randomly distributed across the three regions, showing no clear genetic structure.

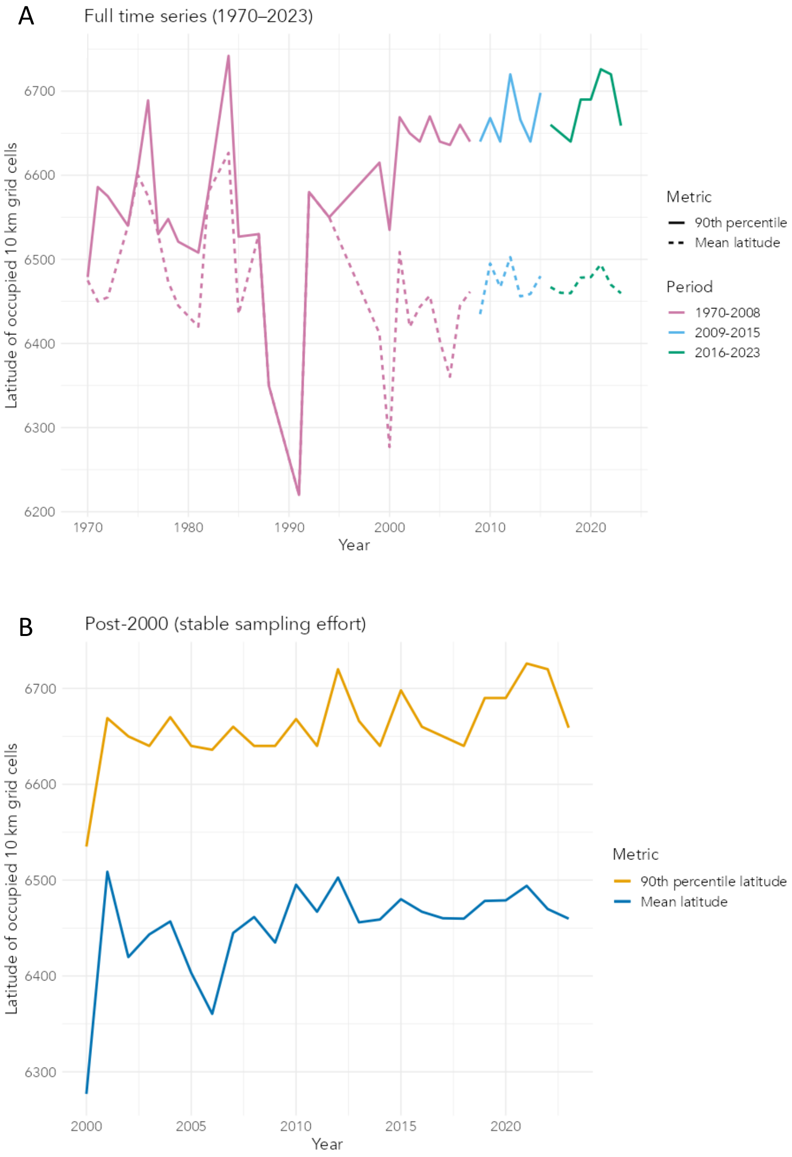

Figure S10. Mean latitude (blue) and 90th percentile latitude (orange) of *B. terrestris* occurrences based on occupied 10 km grid cells. A) full time series (1970–2023); b) subset restricted to 2000–2023.

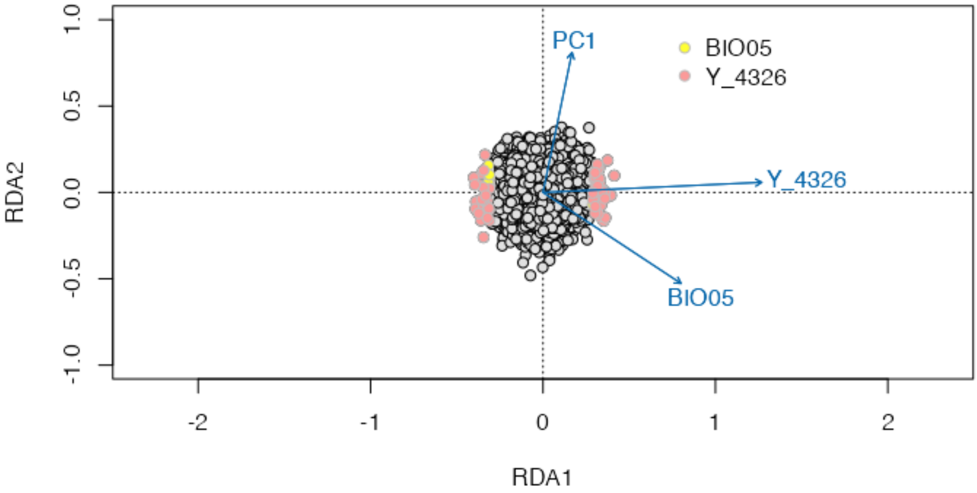

Figure S11. Candidate outlier SNPs (N=69) identified on RDA axis 1 in the simple and complex landscape and temperature study design RDA analysis. BIO05=MaxTempWarmestMonth, Y_4326= latitude.

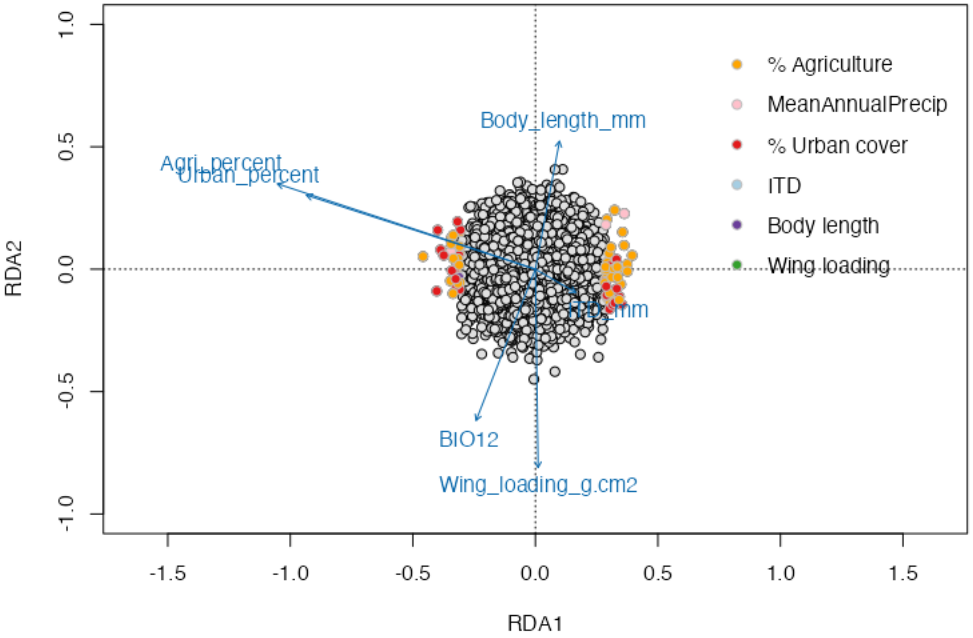

Figure S12. Candidate outlier SNPs (N=76) identified on RDA axis 1 in the other environmental and morphological RDA analysis. Agri_percent= percentage of agricultural land cover, BIO12= MeanAnnualPrecip, mm, Urban_percent= percentage of urban land cover, ITD= inter tegular distance, Body length= body length and Wing loading= wing loading (g/cm^2).
